# Simulation of Cardiac Arrhythmias Using a 2D Heterogeneous Whole Heart Model

**DOI:** 10.3389/fphys.2015.00374

**Published:** 2015-12-21

**Authors:** Minimol Balakrishnan, V. Srinivasa Chakravarthy, Soma Guhathakurta

**Affiliations:** ^1^Department of Biotechnology, Indian Institute of Technology MadrasChennai, India; ^2^Department of Engineering Design, Indian Institute of Technology MadrasChennai, India

**Keywords:** 2D whole heart model, cardiac arrhythmias, AV blocks, WPW syndrome, reduced cell models

## Abstract

Simulation studies of cardiac arrhythmias at the whole heart level with electrocardiogram (ECG) gives an understanding of how the underlying cell and tissue level changes manifest as rhythm disturbances in the ECG. We present a 2D whole heart model (WHM2D) which can accommodate variations at the cellular level and can generate the ECG waveform. It is shown that, by varying cellular-level parameters like the gap junction conductance (GJC), excitability, action potential duration (APD) and frequency of oscillations of the auto-rhythmic cell in WHM2D a large variety of cardiac arrhythmias can be generated including sinus tachycardia, sinus bradycardia, sinus arrhythmia, sinus pause, junctional rhythm, Wolf Parkinson White syndrome and all types of AV conduction blocks. WHM2D includes key components of the electrical conduction system of the heart like the SA (Sino atrial) node cells, fast conducting intranodal pathways, slow conducting atriovenctricular (AV) node, bundle of His cells, Purkinje network, atrial, and ventricular myocardial cells. SA nodal cells, AV nodal cells, bundle of His cells, and Purkinje cells are represented by the Fitzhugh-Nagumo (FN) model which is a reduced model of the Hodgkin-Huxley neuron model. The atrial and ventricular myocardial cells are modeled by the Aliev-Panfilov (AP) two-variable model proposed for cardiac excitation. WHM2D can prove to be a valuable clinical tool for understanding cardiac arrhythmias.

## Introduction

Cardiac arrhythmias are disturbances in the normal cardiac activity manifested in terms of morphological variations in the cardiac rhythm or beat frequency (Gaztañaga et al., 2012). They range from simple, asymptomatic ones to major life threatening arrhythmias which can cause sudden cardiac death (SCD). Globally cardiovascular disease forms nearly 50% of the non-communicable diseases (37 million) and accounts for 17.3 million deaths per year, emerging as the leading global cause of death (Mendis et al., 2011). The number is expected to increase to >23.6 million by 2030 (Mendis et al., 2011; Laslett et al., 2012). Cardiac arrhythmias are a major factor (80%) of SCD, the most fatal ones being ventricular arrhythmias (Mendis et al., 2011). Basic physiological mechanism of each type of cardiac arrhythmia is different and different factors cause the onset of each.

Many whole heart models (ventricular models, atrial models, or whole heart) are designed to simulate the electrocardiogram (ECG) and explain cardiac function in normal and pathological conditions (Van Oosterom, 2001; Potse et al., 2013; Boulakia et al., 2010; Bishop and Plank, 2011; Sovilj et al., 2013, 2014; Casaleggio et al., 2014; Krishnamoorthi et al., 2014). However, there are very few models that show whole heart electrical activity with realistic ECG waveform. A 12 lead ECG of ventricular activity is simulated (Boulakia et al., 2010) based on bidomain approach with heart-torso coupling using a two variable model proposed by Mitchell and Schaeffer (2003). The results are shown in different conditions including heart torso uncoupling, monodomain approximations, different conductivities, and anisotropies. It was concluded that heart-torso uncoupling does not affect the shape of ECG, bidomain equations can be replaced by monodomain approximations (both reduces computational load), but cell heterogeneity and fiber anisotropy affect the shape of ECG waveform (Boulakia et al., 2010). In another bidomain approach, computational complexity is reduced by augmented monodomain approach in which an augmentation layer is defined to have conductivity more close to bath conductivity than to interstitial conductivity and the results are shown to be comparable to those obtained with bidomain equations (Bishop and Plank, 2011).

A 2D model of ventricles is developed with human ventricular cell model proposed by Ten Tusscher and Panfilov (2006) to assess changes in ECG under ischemic conditions. Ischemia is characterized by increase in resting membrane potential and shortening of APD. ECG is computed from the model and shown that ST elevation corresponds to transmural ischemia and ST depression corresponds to endocardial ischemia which correlates with clinical findings (Benson et al., 2008; Lu et al., 2010). Simplified 2D and 3D bidomain models of lesser computational complexity are proposed by defining seven sub regions corresponding to cardiac regions with characteristic cell properties (SAN, atria, AVN, His bundle, bundle branches, Purkinje fibers, and ventricles) and tissue conductivities (Sovilj et al., 2013, 2014). The results produced are comparable in both 2D and 3D simulations with very less computation time for 2D model (6 min for 1 s of cardiac activity). The 2D model shows that variation of APD in ventricles produces QT interval variations (Sovilj et al., 2014) and in 3D model ST segment elevations and depressions are observed for myocardial infarctions (Sovilj et al., 2013). A simple heterogeneous 2D whole heart model based on discrete approach with GJC distribution and APD heterogeneity shows that realistic ECG can be computed with less computational load (Balakrishnan et al., 2014). From the modeling approaches (Boulakia et al., 2010; Balakrishnan et al., 2014; Sovilj et al., 2014) it has been shown that a simplified approach and approximations can give rise to a model with reduced complexity and can explain complex cardiac phenomenon. Since ECG is a non-invasive method to assess the electrical activity of heart, models that can simulate arrhythmias in terms of the associated ECG changes have a significant clinical utility. They offer a clear insight into the mechanisms of arrhythmias and a better interpretation of ECG.

Most of the computational models are developed without the associated ECG simulations to analyze different factors that cause arrhythmia. There are models that explain the role of transmural dispersion of action potential duration (APD) as a cause of reentry (Clayton and Holden, 2004), the role of electrotonic modulation in reentry with finite element approach (Bishop et al., 2013) and myofibroblast density as a cause of arrhythmia in an infarcted heart using an image based model (McDowell et al., 2011). Biophysically detailed atrial models (Kneller et al., 2002; Vigmond et al., 2004; Ridler et al., 2011) can simulate normal and arrhythmic conditions and also response to ablation procedures. Image based models represent the complex geometrical structure of atria and also includes functional aspects such as fiber orientation and APD heterogeneity (Vadakkumpadan et al., 2009; Ridler et al., 2011). 2D tissue models have been proposed to analyze specific arrhythmia conditions like long QT syndrome (Clayton et al., 2001) which can cause the risk of ventricular arrhythmia and SCD. A detailed review of the ventricular and atrial models are given in Trayanova (2012). Most of the existing computational models only explain a few selected types of arrhythmias like atrial fibrillation (Aslanidi et al., 2011), ventricular fibrillation, re-entrant ventricular tachycardia (Clayton and Bishop, 2014) and quite often not in the context of ECG waveform. Furthermore, in most of these models, variation in gap junctional conductance (GJC) is not taken into account. A 1D multicellular model including the SAN (SA node), atrial muscle, and AVN (AV node) is developed with fast and slow pathways to analyze the functions of AVN (Inada et al., 2009). A delay is introduced in AVN for providing adequate time interval between atrial and ventricular contractions. The model (Inada et al., 2009) shows that this is related to the low Na^+^ conductance and poor electrical coupling because of lower GJC (gap junction conductance)in AVN. When the fast pathway is blocked, impulse reaches AV junction through slow pathway which can cause re-entry into the atria as the impulse propagates retrogradely through the fast pathway. The 1D model that consists of both the pathways shows the effect of re-entry by varying the conductance across the different types of cells in AVN (AVN consists of three different types of cells- atrio-nodal, nodal, and nodal-His cells). In a recent computational study of 2D network of myocardial cells it is shown that the variation in GJC inside an ischemic area can generate arrhythmic conditions (Casaleggio et al., 2014). By varying the intercellular and intracellular parameters arrhythmia conditions can be simulated in the model.

Cardiac arrhythmias are classified according their respective causative factors: there are those that are caused by impairment of the autonomic nervous system like normal sinus tachycardia (heart rate (HR) is greater than 100 BPM) (Bauernfeind et al., 1979), sinus bradycardia (HR is lesser than 60 BPM), respiratory sinus arrhythmia (HR is modulated by the respiratory rhythm) (Yasuma and Hayano, 2004), pre-excitation arrhythmia (Wolf-Parkinson-White (WPW) pattern (due to the accessory pathway between atria and ventricles, ventricles are pre-excited) (Douglas and Zipes, 2009), WPW syndrome (through the accessory pathway, bundle of His, and AN node retrograde conduction can occur and this can cause reentry tachycardia) (Sethi et al., 2007), those that are caused by AV conduction blocks (Douglas and Zipes, 2009) (First degree, Second degree Type I (Wenckebach), Type II (Mobitz Type II), Complete heart block) and those that are caused by sinus dysfunction (Strauss et al., 1976) (sinus pause or sinus arrest). In the present study all the above mentioned arrhythmias are successfully simulated.

The size of a single cardiac myocyte is of the order of a few microns while the size of the human heart dimension is approximately 12 cm in length, 8 cm in wide, and 6 cm in thickness (Betts et al., 2013). Approximately 6 × 10^10^ cardiac myocytes are present in an adult human heart. Therefore, computational modeling at cell level by discrete approach requires enormous computational load even for the simulation of 1 s of cardiac activity (Bordas et al., 2009). For analyzing cardiac arrhythmias several seconds of simulations are required and modeling at the resolution of human heart takes extensive computation. In the present study a simplified discrete approach is used in which whole heart is represented by a 2D network of 24000 cells having representative cells from each type and reduced cell models are used to model its dynamics (Balakrishnan et al., 2014). Autorhythmic cells (SAN cells, AVN cells, bundle of His cells and Purkinje cells) of the specialized conduction system are modeled by Fitzhugh-Nagumo two variable model (Fitzhugh, 1961) and (atrial and ventricular) myocardial cells by Aliev-Panfilov two variable model (Aliev and Panfilov, 1996).

Computational models that incorporate structural integrity (atrial and ventricular musculature with specialized conduction system at the whole heart level) and functional parameters including GJC across the cells, APD heterogeneity, variation in the frequency of oscillations (pacing) of the various autorhythmic cells through the specialized conductive system, variation of refractory period (RP) in the generation of various arrhythmias at the whole heart level (with respect to the ECG waveform) are absent. The proposed two-dimensional whole heart model (WHM2D) can explain many of the arrhythmias by varying the GJC across the cells, APD of cells, excitability of the autorhythmic cells, frequency of oscillation of the autorhythmic cells and RP of cardiac cells. The set of simulations include both fatal and non-fatal arrhythmias. This simple 2D model can be used for other types of arrhythmias also by properly selecting the GJC across the cells, APD of the cells, and RP of the cells in the concerned region.

## Methods: a two-dimensional whole heart model (WHM2D)

**Figure 1:**
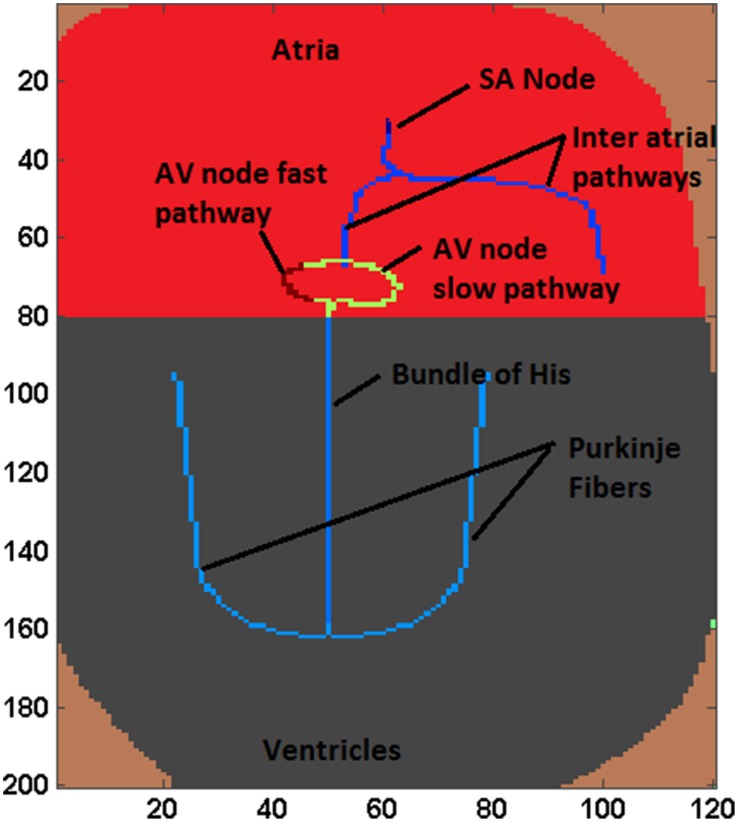
**A simplified schematic of the 2D structure of heart used in the simulation studies**.

The proposed WHM2D, which is based on a simplified representation of the 2D geometry of heart, comprises of all essential cardiac cell types: SA nodal cells, atrial myocytes, fast conducting inter-atrial cells, AV nodal cells, bundle of His cells, Purkinje cells, and ventricular myocytes arranged as shown in the Figure 1. The right side of the atrial region represents the right atrium (RA) and the left side denotes the left atrium (LA). SAN is placed at the center-upper part of the atrium. Two fast conducting inter-atrial pathways are also shown in the Figure 1, one conducting SA nodal signals to the right atrium and the other toward the left atrium. The WHM2D is divided vertically into two regions separated by a dark line (Figure 1): the upper region representing the atria and the lower one representing the ventricles. The low conductance band (dark line) separating atria and ventricles prevent the direct propagation of impulse from the atria to the ventricles other than via the AVN. An impulse from the atria reaches ventricles only via the AVN and the bundle of His and then to the Purkinje fibers. To create asymmetry between the right ventricle and the left ventricle, the bundle fiber is positioned toward the right ventricle (left ventricle is thicker than the right ventricle). GJC between bundle cells to the myocardial cells is kept low to prevent direct spread of the impulse from the bundle cells to the myocardial cells. The GJC between the Purkinje cells and ventricular myocardial cells is high facilitating rapid spread of impulse into the myocardium.

Autorhythmic cells in the SAN produce impulses at the rate of 79 BPM. The impulse spreads through the inter-atrial fibers into the atrial myocardium. Once the impulse reaches the AV junction it is delayed by the low GJC in this area, during which time the signal spreads throughout the atrium. The spread of the impulse in atrial myocardium is from the SAN to the AVN, and then to LA and RA. Subsequently, the signal slowly spreads through the bundle of His positioned in the middle of the ventricles. Once the impulse reaches the Purkinje fibers which are connected to the bundle cells, it propagates along the left and the right branches of the Purkinje network. The GJC between ventricular myocardial cells is also made high ensuring that the impulse spreads through the myocardium at the highest conduction velocity. Ventricular activation spreads from the apex to the base of the heart showing that the ventricular contraction process starts from the apex of the heart. Among Purkinje cells the high GJC is mainly contributed by abundance of connexin protein Cx40 which causes high conductance channels compared to that in ventricular myocardial cells (Kanter et al., 1993). In the WHM2D model also GJC among the Purkinje cells is set to much higher values than that of the ventricular myocardium to obtain a faster conduction and a sharp R wave.

Each cell in the myocardium is connected to six to eleven cells (in atria 6.4 ± 1.7, ventricles 11.3 ± 2.2, Saffitz et al., 1994). In the 2DWH model each cell is connected to its eight adjacent neighbors through GJC. The value of the GJC varies according to the type of the cell to which each cell is connected. A proper distribution of the GJC in WHM2D causes a pattern of activation propagation corresponding to normal sinus rhythm and results in a normal ECG waveform. Differences in conductance values in different directions can account for the fiber orientation and anisotropy present in the cardiac musculature in different regions of the heart (Kanagaratnam et al., 2002). The cells in the specialized conduction system are placed in the WHM2D by analyzing the anatomical and physiological factors. ECG is computed from the model as per the method proposed by Virag et al. (1998) and position of the cells and GJC can be modified by the ECG signal. This makes it possible to perform inverse computation and personalization of ECG.

Different cell types in the WHM2D model are modeled by different low-dimensional cardiac cell models. Oscillatory cells in the cardiac conduction system which include the SA nodal cells, fast conducting atrial cells, AVN, cells of the bundle of His, and Purkinje cells are modeled using Fitzhugh Nagumo (FN) two-variable model (Rocşoreanu et al., 2000). SA nodal cells fire at the highest frequency of 80 BPM, the AVN at 60 BPM; at the end of the Purkinje network the intrinsic frequency reduces to 20 BPM. Atrial myocardial cells and ventricular myocardial cells are represented with the Aliev-Panfilov (A-P) two-variable model proposed for cardiac myocyte (Aliev and Panfilov, 1996). In AVN, excitatory cells are represented with FN model in excitatory mode.

The action potential for a single cell is described using the following general equation

(1) $$\begin{array}{l} C_{m}\frac{dv}{dt}=-\left(I_{ion}+I_{stim}\right) \end{array}$$

where v is the voltage across the cell membrane, *C*_*m*_ is the specific capacitance of the cell membrane, *I*_*ion*_ is the sum of all the individual ionic currents, *I*_*stim*_ is the externally applied stimulus current. In the WHM2D, since each cell is connected to eight neighbors, *I*_*stim*_ is the summation of all individual currents from the eight cells. The individual current depends on the voltage difference and GJC between the cells. This current represents the discrete form of the diffusion current that flows into medium.

(2) $$\begin{array}{l} I_{stim}=\left(V_{i-1,j-1}-V_{i,j}\right)G_{i-1,j-1}+\left(V_{i-1,j}-V_{i,j}\right)G_{i-1,j}\\ +\;\left(V_{i-1,j+1}-V_{i,j}\right)G_{i-1,j+1}+\left(V_{i,j-1}-V_{i,j}\right)G_{i,j-1}\\ +\;\left(V_{i,j+1}-V_{i,j}\right)G_{i,j+1}+\left(V_{i+1,j-1}-V_{i,j}\right)G_{i+1,j-1}\\ +\;\left(V_{i+1,j}-V_{i,j}\right)G_{i+1,j}+\left(V_{i+1,j+1}-V_{i,j}\right)G_{i+1,j+1} \end{array}$$

The equations describing the reduced cell models are given below.

### Reduced models

#### FitzHugh-Nagumo (FN) model

(3) $$\begin{array}{l} dv∕dt=v\left(v-a\right)\left(1-v\right)-w+I_{stim}\\ dw∕dt=b\left(v-\gamma w\right) \end{array}$$

The fast variable *v* models the membrane potential of the cardiac cell and the slow variable *w* models the recovery of the membrane potential. The parameters γ, *a*, and *b* control the behavior of the model (Fitzhugh, 1961; Rocsoreanu and Giurgiţeanu, 2000).

The parameters γ, *a*, and *b* are assumed so that there exists a unique equilibrium point (*v*_*eq*_*, w*_*eq*_) for each cell. Now the equations are modified as follows

(4) $$\begin{array}{l} f\left(v\right)=v\left(v-a\right)\left(1-v\right)\\ dv∕dt=f\left(v+v_{eq}\right)-f\left(v_{eq}\right)-w+I_{stim}\\ dw∕dt=b\left(v-\gamma w\right) \end{array}$$

FN model is used in oscillatory as well as excitable mode by varying the parameter *v*_*eq*_. When *v*_*eq*_ is below the knee of the v nullcline, the model exhibits excitability and when *v*_*eq*_ is above the knee it exhibits oscillations. In cases of premature beats and ectopic foci, excitable cells in the myocardium gains autorhythmic behavior. It has been shown that down regulation of inward rectifying current *I*_*K*1_ induces autorhythmicity in ventricular cells (Miake et al., 2002). In FN model *v*_*eq*_ determines whether the cell is excitable or autorhythmic for a fixed value of *a*. Under pathological conditions, both excitability and autorhythmicity of the cardiac cells can be affected by extrinsic and intrinsic factors. In case of sinus pause or sinus arrest, auto-rhythmicity of SA nodal cells is affected and the cells fail to produce impulse for a short duration of time (Jordan et al., 1978; Gregoratos, 2003). In WHM2D model this is simulated by briefly reducing *v*_*eq*_ of SA nodal cells.

In excitable mode, *I*_*stim*_ represents the stimulation current given for each individual cell. As each cell is connected to its eight adjacent neighbors, if any of the neighbors is excited it can cause an *I*_*stim*_ to flow into the cell. For the oscillatory model, irrespective of the value of *I*_*stim*_, the cell oscillates at the frequency at which the entire structure oscillates. Otherwise it follows the stimulating oscillations as it happens when SAN drives other autorhythmic cells in the specialized conduction system of heart.

#### Aliev-Panfilov (A-P) model

Myocardial cells in the WHM2D model are implemented by the Aliev-Panfilov model proposed for cardiac stimulation.

(5) $$\begin{array}{l} dv∕dt=kv\left(v-a\right)\left(v-1\right)-vw+I\\ dw∕dt=\gamma \left(v,w\right)\left(-w-kv\left(v-a-1\right)\right)\\ \gamma \left(v,w\right)=\gamma_{0}+\frac{\mu_{1}w}{\left(v+\mu_{2}\right)} \end{array}$$

The parameters *k*, γ*, and a* relate to the standard FN model parameters and the parameters μ_1_ and μ_2_ are calibrated according to the appropriate restitution curves (Aliev and Panfilov, 1996). The additional parameter γ_0_ termed as *refractoriness* controls the action potential duration (APD) (Hurtado and Kuhl, 2012).

### Setting the parameters in the atrial region

In the atrial region, APD of the myocardial cells is varied using the refractoriness parameter γ_0_ in A-P model. The longest APD is in the area near the center of the SAN and decreases with increase in distance from SAN center (Spach and Heidlage, 1995). In the model, APD is smoothly varied from the SAN center throughout the atria as defined in Equation (6).

(6) $$\begin{array}{l} \gamma_{0atria}\left(i,j\right)=\gamma_{00a}+\gamma_{01atria}\left(i\right)+\gamma_{02atria}\left(j\right)\\ \gamma_{01atria}\left(i\right)=5\times 10^{-10}\times {\left(40-i\right)}^{4}\\ \gamma_{02atria\backslash }\left(j\right)=10^{-6}-7\times 10^{-11}\times {\left(j-70\right)}^{4}\\ \gamma_{00a}=0.0018 \end{array}$$

γ_00*a*_ represents the longest APD of the atrial myocytes near SAN, γ_01*atria*_(*i*) represents the variation of the refractoriness parameter in the apex-base direction of the atria and γ_02*atria*_(*j*) denotes the variation in the transmural direction. Refractoriness parameter γ_0*atria*_ is inversely proportional to the APD: it is maximum in the region of SAN and decreases with distance from SAN.

### Setting the parameters in the ventricular region

From the simulation studies it was understood that in order to obtain a smooth T wave, the repolarization should begin simultaneously from all parts of the ventricular musculature that is from the endocardium, epicardium, base, and the apex. The regions that are depolarized last are the epicardium and apex regions. If repolarization must start simultaneously in all regions, the APD of the apex and epicardium must be smaller than that of the epicardium and base regions. For achieving this, the APD is modified by three parameters: the maximum APD duration, transmural variation and apex-base variation. γ_0*ventr*_ is varied as a function of position of the cell in the matrix as given in Equation (7).

(7) $$\begin{array}{l} \gamma_{0ventr}i\left(i,j\right)=\gamma_{00v}+\gamma_{01ventr}\left(i\right)+\gamma_{02ventr}\left(j\right)\\ \gamma_{01ventr}\left(i\right)=3\times 10^{-10}\times {\left(160-i\right)}^{4}\\ \gamma_{02ventr}\left(j\right)=10^{-6}-7\times 10^{-14}\times {\left(j-50\right)}^{6}\\ \gamma_{00v}=0.0015; \end{array}$$

γ_00*v*_ is the maximum APD of the ventricular myocytes, γ_01*ventr*_(*i*) is the apex-base variation and γ_02*ventr*_(*j*) is the transmural variation in ventricles.

**Figure 2:**
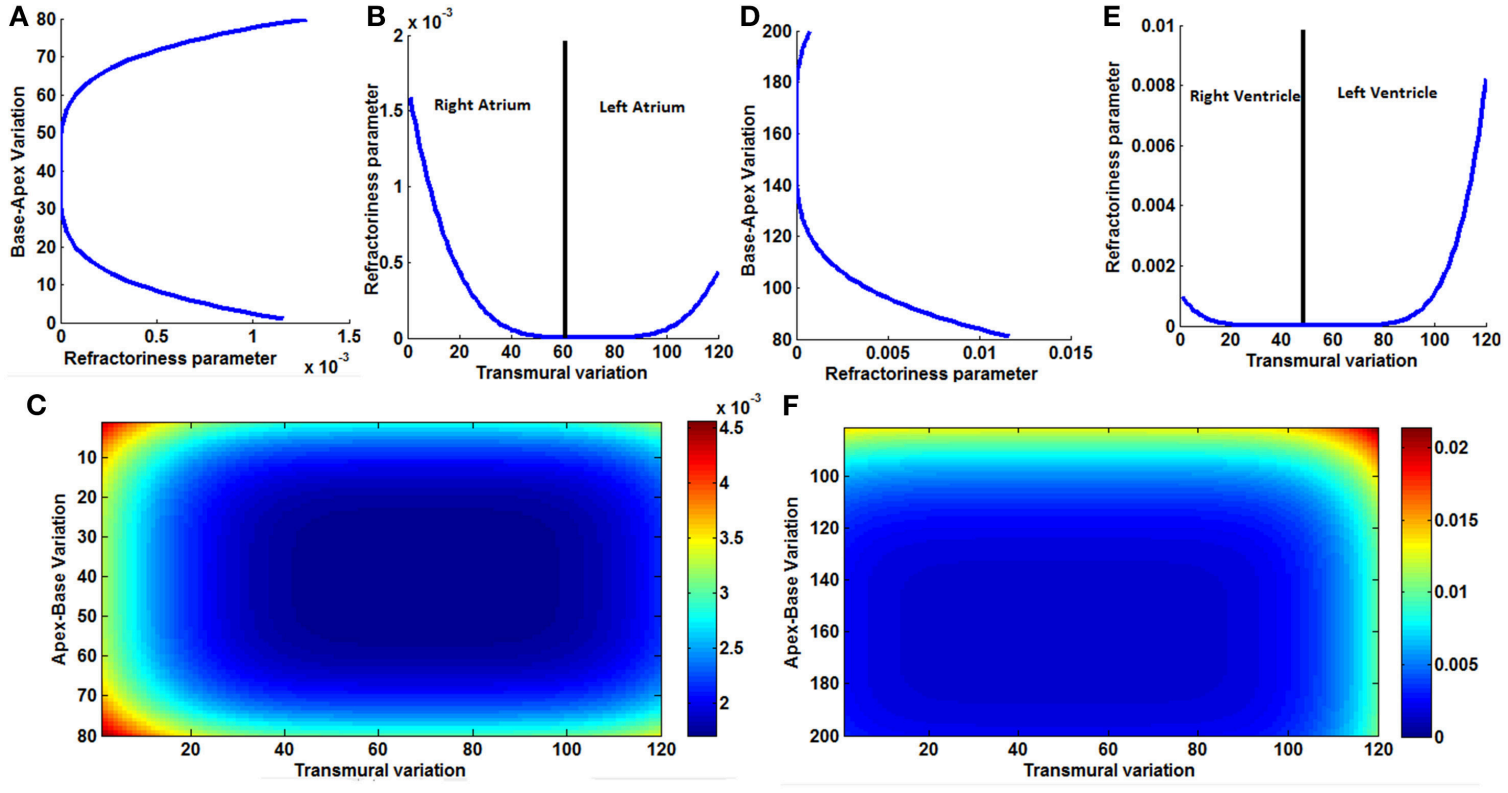
**(A)** Variation of the refractoriness in atria along the base-apex direction, **(B)** variation of the refractoriness in atria along the transmural direction, across the entire wall of the atrial musculature; it is not symmetric between the right atrium and left atrium. **(C)** 2D Variation of refractoriness in atria. **(D)** Variation of the refractoriness in ventricles along the base-apex direction. **(E)** Variation of the refractoriness in ventricle along the transmural direction, across the entire wall of the ventricular musculature; it is not symmetric as left ventricle is thicker than right ventricle. **(F)** 2D Variation of refractoriness in ventricles.

A comparison of the variation of refractoriness parameter in apex-base direction, transmural direction and the 2D distribution in the model for atria and ventricles are shown in Figure 2. As the spread of the repolarization wave is different for atria and ventricles, the 2D distribution of the aforementioned parameters should also be different in atria and ventricles. The maximum variation is less for atria compared to ventricles. This is because Ta (atrial repolarization) wave for atria has negative polarity with lesser amplitude than P wave, but the T wave (ventricular repolarization wave) has positive polarity and requires large variation of APD to initiate repolarization from all regions of ventricles.

### Computation of ECG from the WHM2D

**Figure 3:**
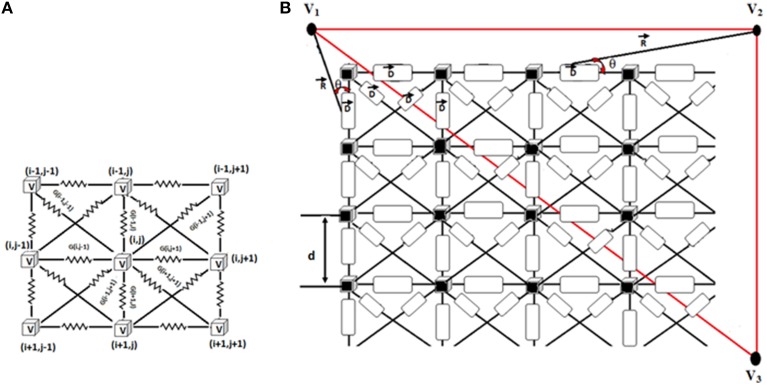
**(A)** Connection of each cell in the 2D matrix. Each cell is connected to eight neighbors. 2D cell network size is 200 × 120. **(B)** Measurement of ECG signal from the 2D network. R shows the position vector between the computed point and the source. θ denotes the angle between the position vector R and the dipole D.

ECG signal is computed from the WHM2D by the method described by Virag et al. (1998) as shown in Figure 3. In the 2D network of cardiac cells, each pair of adjacent cells forms an electric dipole of length “*d*” and current density “*I*” (Δ*V*_*m*_ × *G*). The potential recorded at V_1_, V_2_, or V_3_ is equal to the summation of the contribution of each dipole in the network.

The potential projected from a dipole to the measuring point is calculated as follows (Equation 8).

(8) $$\begin{array}{l} V\left(i,j\right)=\frac{\left(\Delta V_{m}\times G\right)\times d^{2}\times \cos\left(\theta \right)}{4\pi R^{2}} \end{array}$$

Where Δ*V*_*m*_ is the potential between two adjacent cells which forms a dipole, *G* is the conductance between them, *d* is the length of the dipole, and θ represents the angle between the position vector *R* and the dipole *D*. The contributions *V*(*i,j*), of each dipole in the vertical, horizontal and two diagonal directions are calculated (Equation 9).

(9) $$\begin{array}{l} V\left(k\right)=\overset{200}{\underset{i=1}{\sum }}\overset{120}{\underset{j=1}{\sum }}V_{ver}\left(i,j\right)+V_{hor}\left(i,j\right)+V_{diag1}\left(i,j\right)+V_{diag2}\left(i,j\right) \end{array}$$

The standard ECG limb leads and augmented leads can be computed from the summated voltages at three points (Virag et al., 1998) as shown in Equation (10).

(10) $$\begin{array}{l} LeadI=V2-V1;LeadII=V3-V1;LeadIII=V3-V2\\ Vo=\frac{\left(V1+V2+V3\right)}{3}\\ aV_{R}=\frac{3\times \left(V_{1}-V_{o}\right)}{2};\\ aV_{L}=\frac{3\times \left(V_{2}-V_{o}\right)}{2};\\ aV_{F}=\frac{3\times \left(V_{3}-V_{o}\right)}{2} \end{array}$$

## Results

### Normal ECG

**Figure 4:**
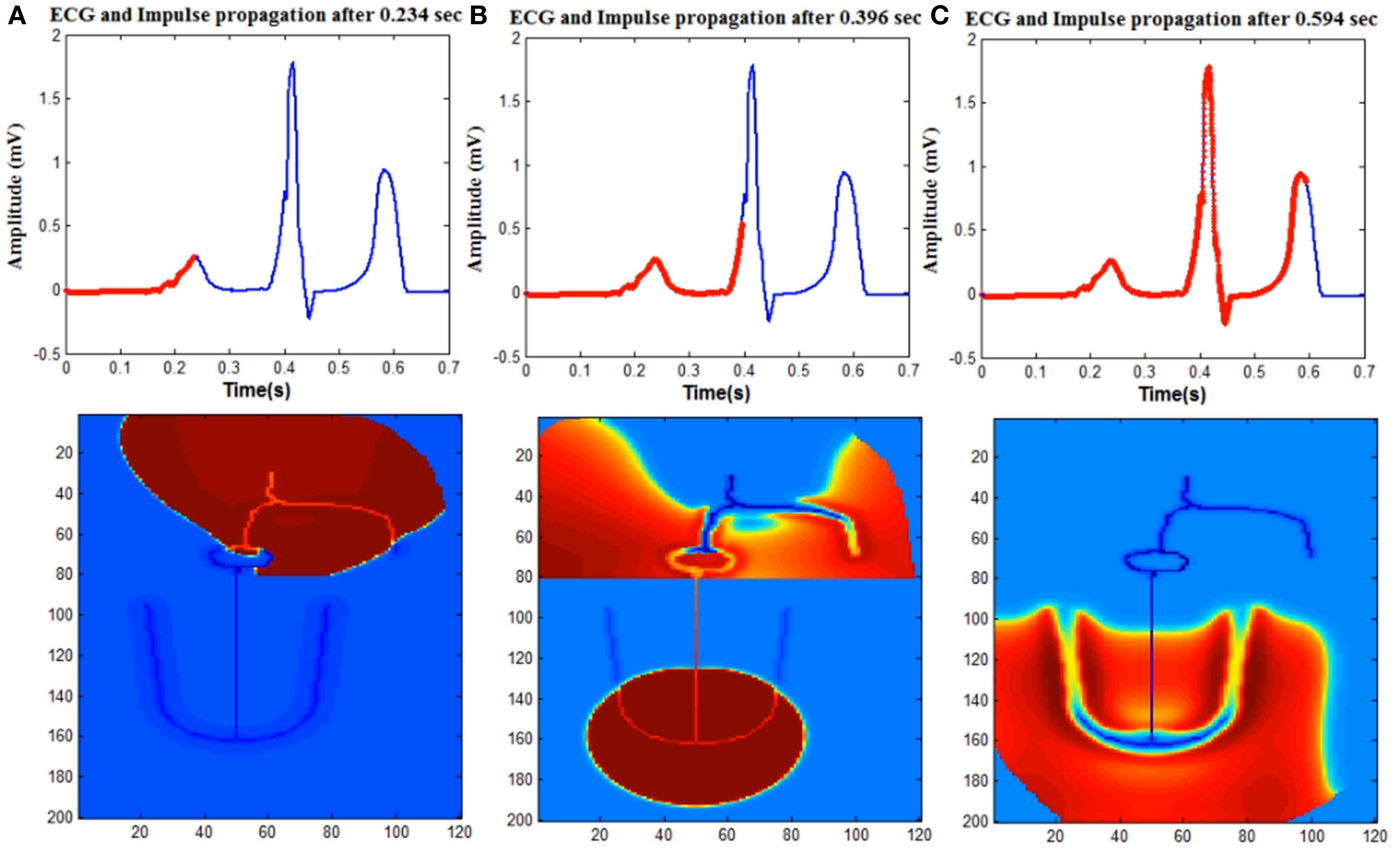
**Upper trace shows ECG signal generated from the model and lower one shows the spread of impulse in the WHM2D**. The red line on the ECG signal shows the instance of the spread of wave propagation in the 2D model **(A)** atrial depolarization (P wave), **(B)** ventricular depolarization (QRS complex). Atrial repolarization is happening at the same time and obscured by the high amplitude QRS complex. **(C)** Ventricular repolarization (T wave).

Normal ECG is initiated by the spontaneous impulse produced by the autorhythmic cells in the SAN at the rate of 73 BPM. This impulse depolarizes the cells in atrial myocardium through the internodal pathways and reaches AVN. Spread of the depolarization impulse in the atrial myocardium results in P wave. The impulse is delayed in the AVN and bundle of His, which produces the delay (PR interval) between atrial depolarization and ventricular depolarization. The only electrical connection between atria and ventricles is through AVN. From AVN the impulse passes through the bundle of His cells and reaches Purkinje fibers which branches to the left and right ventricles. From the Purkinje cells, the impulse spreads to the ventricular myocardium (Figure 4).

WHM2D consists of 24000 cells (200 × 120 matrix) out of which 9600 cells (80 × 120) are allotted for the atria (vertically divided equally into right atrium and left atrium) and rest for the ventricles. Within the atria, SA nodal cells, cells in the inter-nodal pathways and AVN are connected by high GJC. Atrial myocardial cells are connected to the cells in the internodal pathways. The impulse spreads asymmetrically in right and left atria as the fiber orientation and distribution of GJC is different. The distribution of the connexin proteins Cx40 and Cx43 is different in the LA and RA regions. It has been shown that the right atrial conduction velocity is inversely proportional to the ratio Cx40/(Cx40 + Cx43), but linearly related to Cx43/(Cx40 + Cx43) (Kanagaratnam et al., 2002). Even though the density of Cx40, which has a high unitary conductance, is higher in RA than in LA (Vozzi et al., 1999), the conduction velocity is less in RA. This is because in RA, the gap junctions form heteromeric gap junction channels, comprising Cx40 and Cx43, a channel type that has low unitary conductance (Kanagaratnam et al., 2002). There are also differences in fiber orientation between the RA and LA (Wang et al., 1995; Valderrábano, 2007). This causes the spread of the impulse more in horizontal direction in the LA than in RA (Figure 4A).

Atrial repolarization (Ta) wave is usually not seen in the ECG waveform as the repolarization process coincides with the time of ventricular depolarization. The small repolarization wave is obscured in the presence of higher amplitude QRS complex. But it can be observed in patients with arrhythmias when the impulse from the atria is completely blocked from entering the ventricles. The amplitude of the Ta wave is 1/10th of the P wave and the phase of the Ta wave is opposite to that of the P wave (Holmqvist et al., 2009). In the simulation, a moderate APD variation as per Equation (6) is introduced in the atrial musculature so that the amplitude of the wave is reduced and the polarity is made opposite to that of the P wave. The existence of heterogeneity of APD is well-established in ventricles (Antzelevitch et al., 1999). In atrial musculature also heterogeneity is observed and has been analyzed for atrial reentry and fibrillation studies. The longest APDs are found in the area where the atrial excitation is initiated (SAN), while there is progressive shortening of the APD with increasing distance from the sinus node. The authors also state that the variation of the APD must be smooth without discontinuities, since discontinuities cause atrial fibrillation and reentry arrhythmias (Spach and Dolber, 1986). The refractoriness parameter γ_0_ of the atrial myocyte is smoothly varied from the SAN across the boundaries (Hurtado and Kuhl, 2012) as per Equation (6). The 2D distribution of the refractoriness parameter γ_0_ is shown in Figure 2A.

The lower 120 × 120 region of the WHM2D represents the ventricles (Figure 1). The impulse reaches the bundle of His cells through the AVN. The other end of the bundle fibers are connected to the Purkinje fibers. In the depolarization process, the impulse travels down through the bundle of His fibers in the septum to the Purkinje fibers. The Purkinje network branches to the left and the right, penetrating through the ventricular myocardium. Once the impulse reaches the Purkinje network, it starts spreading into the myocardium. Thus, the propagation starts from the apex of the heart and spreads upwards to the base region instantaneously as the GJC values are higher among the Purkinje cells than between the myocytes and Purkinje cells. This results in a sharp R peak. The S wave of the QRS complex is produced because of the asymmetry between the right and left ventricles (Figure 4B).

Wilson et al. (1931) hypothesized that concordance of the polarity of T wave with R wave may be explained by assuming that at least in some part of the ventricles the depolarization and repolarization waves travel in opposite directions. Electro-physiological studies (Antzelevitch et al., 1999) show that ventricular myocardium is not homogeneous and that there exists at least three types of ventricular cells,—epicardial, mid myocardial, and endocardial cells,—with different values of APD. Noble et al (Noble and Cohen, 1978) showed that the APD from tissue slices dissected from the apex is longer than those from the base of the sheep ventricle. They concluded that such a difference could account for a positive polarity of the T wave in mammals. These studies showed that there is heterogeneity in APD in the apex- base direction and transmural (from epicardium to endocardium) direction. The contribution of each of these heterogeneities to the positive T wave is not clear.

From the simulation studies it was understood that, for obtaining a smooth T-wave, the repolarization should begin simultaneously from all parts of the ventricular musculature—endocardium, epicardium, base, and apex. This is made possible by the APD heterogeneity implemented in the model as per Equation (7). The regions that are depolarized last are the epicardium and base regions, compared to endocardium and apex regions, as they are far away from the site of initiation of depolarization. For repolarization to start simultaneously in all regions, the APD at the base and epicardium must be shorter than that at the endocardium and apex regions (Figure 4C).

The amplitude and duration QRS complex is determined by the GJC among the ventricular cells. S wave in the QRS complex occurs because of the asymmetry between the left and the right ventricle. PR interval depends on the GJC of the AVN and bundle cells, also on the duration of the APD of the atrial myocardium. ST interval is determined by the maximum APD of the ventricular myocardial cells. The positive polarity and the shape of the T wave depend on the dispersion of APD heterogeneity of the ventricular myocardial cells. Simulation of normal ECG signal and corresponding propagation in the 2D model is given in Supplementary Material Video 1.

### Cardiac arrhythmias

The following arrhythmias are simulated in the present model: normal sinus tachycardia, sinus bradycardia, respiratory sinus arrhythmia, Wolf-Parkinson-White (WPW) pattern, WPW syndrome, first degree block, second degree Type I (Wenckebach) block, Type II (Mobitz Type II) block, complete heart block and sinus pause.

Variations in HR can be caused by neural, chemical, hormonal modulations in the body and prominently by the influence of the autonomous nervous system (ANS) (Malliani, 2012). Normal sinus tachycardia, sinus bradycardia, and respiratory sinus arrhythmia can be considered as the impairment of the ANS which regulates HR by balancing two opposing systems: sympathetic system which stimulates the heart and parasympathetic system which inhibits it. Impulse from ANS reaches the heart through the pre- ganglionic nerve fiber which synapses with a secondary neuron within the ganglion and sends impulses to the cardiac cells through the post-ganglionic nerve fiber which delivers impulses directly to the cardiac cells (Thomas, 2011). The effect of parasympathetic stimulation through the cholinergic receptors is to reduce the rate of SAN pacing, slow down the conduction at AVN, increase the APD of AVN cells and reduce the contractility of the excitable myocytes. Sympathetic system releases norepinephrine which activates the adrenergic receptors (α_1_ or β_1_), accelerates SAN pacing, increases conduction velocity and shortens APD of the AVN cells (Levy, 1984; Dubin, 2003).

#### Normal sinus tachycardia

HR in the normal resting state can be considered as a prognostic factor (Fox et al., 2007; Gopinathannair and Olshansky, 2009) and across the animal species high HR is associated with increased mortality (Levine, 1997). Sinus tachycardia (ST), a clinically common condition, is generally defined as the arrhythmic condition when the HR is greater than 100 BPM. Broadly it can be classified into normal sinus tachycardia (NST) and andinappropriate sinus tachycardia (IST). NST has an underlying cause that may be of physiologic, pathologic, and/or pharmacologic origin. IST on the other hand, denotes a breakdown in the mechanism that regulates tachycardia (Yusuf and Camm, 2005). IST can sometimes be associated with upright posture, called the postural orthostatic tachycardia syndrome (POTS) or sinus node reentry tachycardia (SNRT). The exact cause of IST is not clear. For example, it is not known whether it is connected to abnormality in SAN or abnormal autonomic function (Bauernfeind et al., 1979; Nwazue et al., 2014).

#### Normal sinus bradycardia

Sinus bradycardia is the condition when the heart rate is less than 60 BPM. If it is asymptomatic it is considered as a sign of physical fitness. Otherwise, symptomatic sinus bradycardia is a life threatening condition and needs medical care (Dreifus et al., 1991). SAN area is densely innervated by the post-ganglionic cholinergic and adrenergic fibers and therefore ANS stimulation can alter the rate of pacing of SA nodal cells (Pauza et al., 2000). In the resting condition, HR is governed by the parasympathetic system. Vagus nerve activation can inhibit sympathetic activation at the pre-synaptic level (Olshansky et al., 2008), and can reduce SA nodal pacing rate by direct hyperpolarization. Certain studies show that vagally mediated sinus bradycardia can reduce the development of fatal ventricular arrhythmias in the absence of hypotension as the cell irritability is reduced by increasing the refractory period and threshold for electrically induced ventricular fibrillation (Myers et al., 1974).

**Figure 5:**
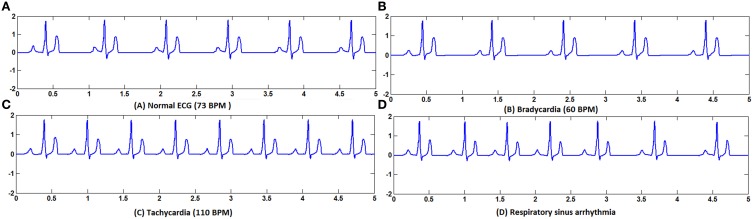
**(A)** Normal ECG, **(B)** Bradycardia simulated by decreasing the frequency of oscillations of the pacemaker cells, **(C)** Tachycardia simulated by increasing the frequency of oscillations of the pacemaker cells, **(D)** Respiratory sinus arrhythmia simulated by modulating the frequency of the pacemaker cells with the respiratory rhythm.

Tachycardia and bradycardia arrhythmias are simulated by varying the parameter *b* which determines the frequency of the FN cells in the specialized conduction system. All the pacemaker cells in the heart are innervated by sympathetic and parasympathetic fibers and the chronotropic effect is different in each type (Jones et al., 1978). In order to simulate tachycardia and bradycardia conditions, increasing or decreasing the SA nodal pacing rate alone does not vary the overall heart rate. Corresponding changes must be made in the pacing rates of subsidiary pacemaker cells also to produce the tachycardia and bradycardia arrhythmias. In WHM2D model sinus bradycardia is implemented by decreasing the pacing rate of the SA nodal cells and increasing the APD of AV nodal and other autorhythmic cells. Sinus tachycardia is implemented by increasing the rate of the SA nodal pacing and decreasing the APD of the AV nodal cells (Figure 5).

The modifications of pacing rates for the autorhythmic cells in the conduction system for tachycardia and bradycardia are shown in the Table 1.

**Table 1:** **Parameters chosen for simulating ECG (normal), sinus tachycardia and sinus bradycardia**.

**Parameters used**	**Normal ECG**	**Sinus tachycardia**	**Sinus bradycardia**
Heart Rate	73 BPM	110 BPM	60 BPM
SA nodal frequency (Highest)	79 BPM	115 BPM	64 BPM
AV nodal frequency (Highest)	65 BPM	80 BPM	56 BPM
Bundleof His (Highest)	63 BPM	60 BPM	43 BPM
Purkinje fibers (Highest)	53 BPM	49 BPM	39 BPM
Atrial conductance	0.004 μS	0.005 μS	0.003 μS
Ventricular conductance	0.08 μS	0.08 μS	0.08 μS
AV conductance	0.1 μS	0.2 μS	0.08 μS

#### Respiratory sinus arrhythmia (RSA)

In RSA the vagal control is withdrawn during the inspiration phase; sympathetic control becomes more prominent during that phase which increases the HR. During the expiration phase, vagal control is reinstated and the rate is back to normal (Yasuma and Hayano, 2004). RSA is a cardio-respiratory phenomenon where the heart rate fluctuation depends on respiratory frequency and depth of ventilation (Hirsch and Bishop, 1981). Central nervous system controls the heart rate by the complex, mutually opposing interaction between sympathetic and parasympathetic systems. The pacing rate of SAN is determined by the interaction between these two systems. Respiratory rhythm modulates the activity of cardiac vagal pre-ganglionic neurons. Inflation of lungs during the inspiratory phase may inhibit the cardiac vagal efferent nerve fibers which carry information from CNS to cardiac cells (Horner et al., 1995). There are different explanations for the occurrence of RSA. It is shown that the pulmonary gas exchange is increased by RSA as increased HR during inspiration increases alveolar ventilation and capillary perfusion rate (Hayano et al., 1996). Many studies cannot confirm the specificity of vagal tone irrespective of sympathetic activity as RSA magnitude is also affected by beta-adrenergic tone (Grossman and Taylor, 2007). In spite of all the controversies, RSA is used as a non-invasive measure of vagal tone to assess the complexities of autonomous nervous system and heart rate variability (HRV) studies. The exact origin of RSA is still debated with many unresolved issues including the interaction of respiratory and circulatory centers and the role of other parts of the brain in RSA (Yasuma and Hayano, 2004).

In order to simulate RSA in the 2D model, the frequency parameter of the SA nodal cells is modulated by the respiratory frequency (*f* = 0.2 Hz). The parameter B which determines the pacing frequency of SAN is defined as,

(11) $$\begin{array}{l} B=b+\sin\left(2\pi ft\right) \end{array}$$

*b*-parameter determining the frequency of SAN (73 BPM)

*f* = 0.2 Hz—respiratory frequency

**Figure 6:**
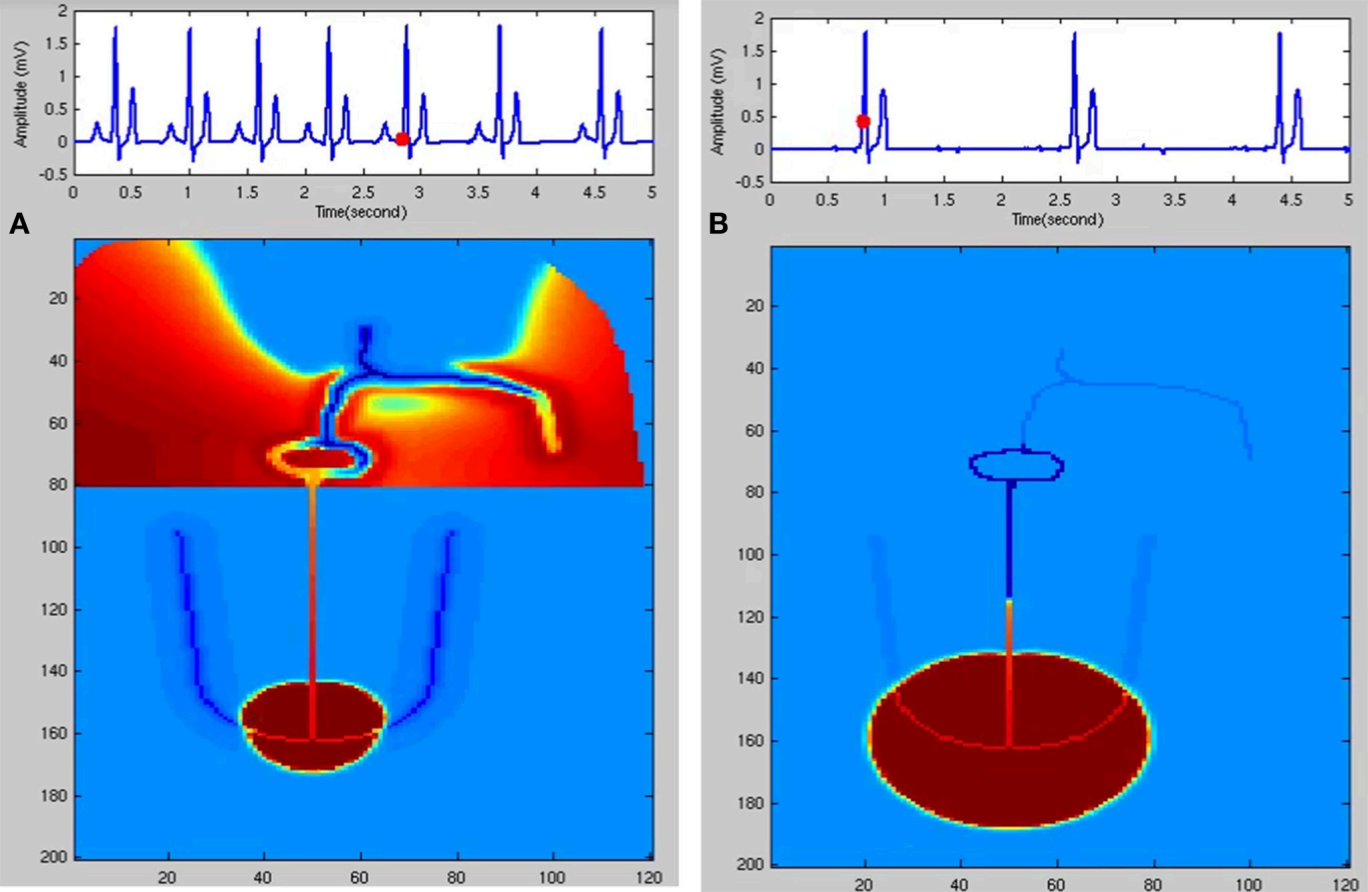
**(A)** Respiratory sinus arrhythmia. SA nodal frequency is modulated by the respiratory rhythm (0.2 Hz). **(B)** Junctional Rhythm with no P wave. SAN is dysfunctional and the atrial cells are not stimulated. A lower rate rhythm produced from the AV junction stimulates the ventricles and QRS complex is produced.

The variation in heart rate (HRV) is an indirect measure of the function of ANS. If an appropriate model of ANS is developed, it can be used to couple with the WHM2D to analyze various aspects of ANS. ECG simulated is shown in Figure 5D and the corresponding spread of the impulse is shown in Figure 6A. Simulation of respiratory sinus arrhythmia and corresponding propagation in the 2D whole heart model is given in Supplementary Material Video 2.

#### Sinus pause

Due to its higher firing rate, SAN occupies the privileged position as the pacemaker of heart, though there are autorhythmic cells in AVN and Purkinje network also. The automaticity of cells in the AVN and Purkinje fibers is suppressed by the overdrive mechanism (Issa et al., 2012). These cells are depolarized much faster than their intrinsic rate as SAN drives them (Mangoni and Nargeot, 2008). Because of this overdrive mechanism there occurs a delay before these cells take up the role of pacemaker of heart in the event of a missed impulse from the SAN. Sinus node dysfunction (SND) is a common disorder that can range from sinus bradycardia to sinus arrest (complete standstill of SAN function). Usually this is diagnosed in elderly patients and the symptoms can be shortness of breath, palpitations, or syncope (Gregoratos, 2003). The causes of SND can be intrinsic, where the electrophysiological properties of SA nodal cells are altered, or extrinsic, where disturbances in the ANS which controls the pacing properties play a role (Strauss et al., 1976; Jordan et al., 1978), as well as antiarrhythmic drugs (Yeh et al., 1991; Mallet, 2004). In most cases of SND, atrial tachyarrhythmias are associated with SND and it is not clear whether atrial substrate remodeling near the SA nodal region can down regulate the channel properties so as to reduce the automaticity of SA nodal cells (Chang et al., 2013).

Sinus pause or arrest is mainly caused by the SND in which the excitability functions of the SA nodal cells are suppressed. The ion channels involved in the pacemaking property of the SA nodal cells can be affected by genetic defects, aging, medication, and influence of ANS. SA nodal remodeling also happens due to atrial tachyarrhythmia, surgical trauma, and myocardial infarction (Wolbrette and Naccarelli, 1998). Sinus pause is manifested in ECG as missing ECG waves, which can last for <1 s or for longer durations leading to weakness and syncope. If the pause exceeds 3 s, the patient needs pacemaker support and it comes under class IIa recommendation for permanent pacing under SND (Epstein et al., 2008). After sinus pause, a junctional or ventricular escape rhythm may occur. Sinus pause, if asymptomatic, can be ignored but if symptomatic it may be because of the degeneration of SA nodal cells, myocardial infarction, antiarrhythmic drugs (beta-blockers calcium channel blockers, digitalis), high blood potassium (hyperkalemia), low-level thyroid hormone in the blood (hypothyroidism), sleep apnea and thorough investigation is required to find the exact cause (Gregoratos, 2003).

**Figure 7:**
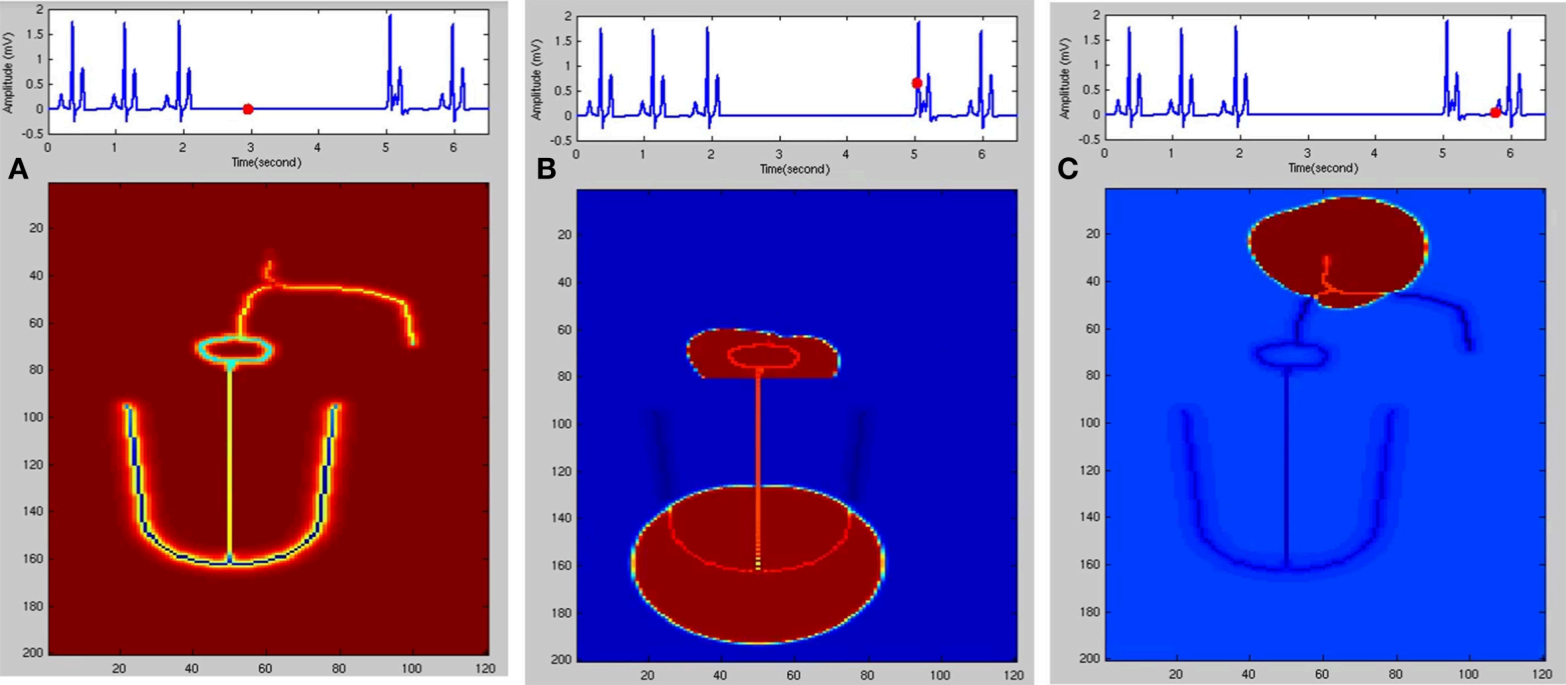
**Sinus Pause**. Figures in the top row denote ECG waveforms, while the images in the bottom row represent the corresponding state of activation of the 2DWH model. **(A)** The red dot shown in the ECG waveform indicates the paused state of the model depicted in the bottom row. The corresponding image below shows that there is no activity in the model **(B)** after sinus pause a junctional escape occurs which drives the ventricles in the anterograde pathway and the atriainretrograde pathway. Since atrial activity is occurring during ventricular depolarization, P wave is obscured in QRS complex. **(C)** After junctional escape a normal sinus rhythm originates from the SAN.

In the FN model the excitable parameter *v*_*eq*_ determines whether the cell is in excitable or autorhythmic mode (Equation 4). If *v*_*eq*_ is greater than 0.16 the cell behaves as an oscillatory cell producing its own oscillations and when *v*_*eq*_ is less than 0.16 it requires external stimulation to produce an action potential. In sinus pause the excitable parameter *v*_*eq*_ is reduced below 0.16 for duration of 0.3 s by modulating *v*_*eq*_ by a square wave of 0.15 Hz. This can simulate sinus pause in the WHM2D as shown in Figure 7. Simulation of sinus pause and corresponding propagation in the 2D whole heart model is given in Supplementary Material Video 3.

#### AV conduction blocks

Atrio-ventricular (AV) node is the only electrical connection between the atria and ventricles through which the impulse from the atria can pass into the ventricles (Miyazaki, 2014). Anatomical mapping of the conduction system by Tawara (2000) in various mammalian heart studies revealed the exact position of the AVN, bundle of His and its associated branching. Tawara (2000) described AVN as a compact spindle shaped network of cells arranged in a node which is connected to the His bundle at the descending end, while the other end is connected to the atrial musculature. AV node consists of three regions, atrio-nodal (AN) region, which is connected to the atrial musculature, central nodal (N) region and Nodal-His bundle (NH) region, which connects to the His bundle. N and NH regions contain ovoid cells which possess automaticity and allow the AVN to be the backup pacemaker of the heart when SA node fails to produce impulses at the normal heart rate. As per the microelectrode recordings from the rabbit AV junction, APD increases from the AN to the NH region (Anderson et al., 1974).

Physiologically, AV junction plays important roles in the normal functioning of the heart. It works as a delay unit, where the impulse is delayed before reaching the ventricles allowing enough time for the ventricles to get filled before the systole ends. In the absence of SA nodal impulse, the AVN takes up the pacemaker function of the heart, providing rhythmic pulses at the rate of 60 BPM. In case of atrial fibrillation, atrial flutter, or supraventricular tachycardia, where the atrial contractions are at a much higher rate (> 150 BPM) than normal, AVN acts as a filter providing concealed conduction where only few impulses are passed into the ventricles, preventing ventricles from beating at the same rate as atria. In case of abnormal function, AVN produces different degrees and types of AV block and AVN re-entrant tachycardia (AVNRT). Re-entry circuitry is mainly because of the existence of a slow and a fast pathway of conduction in the AV junction. PR interval in the ECG signal denotes the delay between the atrial and ventricular contractions. If it exceeds 0.2 s the condition is termed as first degree block and it prolongs further for second degree blocks with dropped beats. In second degree, there are two subtypes of blocks: type 1 (Wenckebach) and type II (Mobitz). Third degree blocks are characterized by total blockage of impulse from atria to ventricles. Many functional and mathematical models (Heethaar et al., 1973; Jørgensen et al., 2002; Inada et al., 2009; Climent et al., 2011) have been proposed to explain the concealed conduction in AVN. But computational models that describe the structure and electrophysiological properties of these cells, and explain the contributions of AVN to the ECG signal are nearly absent.

##### First degree block

The delay between the atrial and ventricular contraction is assessed by the PR interval of the ECG waveform. P wave is produced by the spread of impulse throughout the atrial myocardium. The cardiac impulse is delayed in the AVN and bundle of His before reaching the ventricular musculature. PR interval is contributed by conduction time from sinus node to ventricles which includes the time taken for the spread of impulse in the atria, the delay in AVN and bundle of His. In normal functioning of the heart, PR interval is between 0.12 and 0.2 s. If the PR interval is greater than 0.2 s with no failure in AV conduction, the condition is termed as first degree AV block (Barold et al., 2006). This prolongation of the PR interval is fixed throughout the ECG recordings. The causes for first degree AV block with normal QRS complex can be conduction delay in the intra-atrial pathways, AVN, bundle of His, or in Infra-Hisian system, but in the majority of cases it results from atrial or AVN conduction delay (Graybiel et al., 1944). The word “block” is a misnomer since there is only a delay in conduction, and not a total block. This condition is therefore considered to be a benign condition if PR interval is <0.03 s (Perlman et al., 1971). But over long periods it has been shown that first degree block can be a sign of clinical prognosis (Holmqvist and Daubert, 2013) as different studies proved that it can lead to AF or higher degrees of block. Patients with PR interval >0.3 s with a hemodynamic compromise is recommended for pacemaker implantation (Epstein et al., 2008; Barold and Herweg, 2012). Electrophysiological recording of ventricular pulses after atrial stimulations show that first degree block is mainly due to conduction delay in AVN (Damato et al., 1969; Rosen et al., 1971).

In the WHM2D, AVN is composed of two types of cells, oscillatory cells that pace at 60 BPM which set the pace in the absence of SA nodal pacemakingaction and excitable cells. The APD of the AV nodal cells has a positive gradientfrom the AN region to the NH region (Anderson et al., 1974). The GJC of AV cells to the neighboring atrial musculature is made low to make sure that a delay is produced in the AVN and conduction is not affected by the stimulation from adjacent atrial myocytes. Different types of conduction blocks can occur in AVN: first degree block where the PR interval is greater than 0.2 s, second degree block (of two types: type I and type II) and third degree block. Delay between atrial and ventricular contractions can be varied by varying the GJC in the AV nodal and bundle of His cells. Physiologically the absence of connexin40 is reported to cause conduction blocks in AN node and bundle of His (Jansen et al., 2010). The variation of GJC in AVN and bundle of His and the corresponding change in AV delay (PR interval) in shown in the Table 2. Compared to reduction of GJC in AVN, reduction in bundle of His is found to cause more variations in PR interval (Schrickel et al., 2009).

**Table 2:** **PR interval variations corresponding to the variations in GJC [micro Siemens (μS)] among AV nodal cells and bundle of His cells**.

**Gap Junction conductance in AVN (μS)**	**Gap Junction conductance in bundle of His (μS)**	**PR interval (s)**
0.09	0.9	0.18
0.08	0.9	0.183
0.07	0.9	0.186
0.06	0.9	0.202
0.05	0.9	0.226
0.04	0.9	0.237
0.04	0.6	0.279
0.01	0.5	0.307

##### Second degree block

Second degree AV block can be classified into Mobitz types I and II. Mobitz type I is also called as Wenckebach block which is characterized by progressive PR prolongation, finally resulting in a missed beat. The ratio of original and conducted beats is X:X-1 i.e., out of X atrial contractions (P waves) only X-1 ventricular contractions (QRS complexes) occur. PR interval remains constant in Mobitz type II block (Zipes, 1979) and the ratio of atrial pulses to conducted beats from atria is X:1 i.e., out of X atrial contractions (P waves) only one ventricular contraction (QRS complexes) occurs. In both conditions, atrial impulse is blocked in different ratios. If the condition is prolonged for a longer duration finally it can result in third degree block where the atrial pulses are totally blocked from entering the ventricular conduction system.

*Wenckebach Phenomenon (Mobitz Type I)*. Second degree type 1 AV block was observed by Karel F. Wenckebach in 1899 from the radial pulse even before the advent of ECG or the discovery of SAN and AVN (Upshaw and Silverman, 1999). Type 1 second degree block, which is termed as Wenckebach block, can occur at any anatomical junction in the cardiac musculature where there is delay normally present or potentially possible (Decherd and Ruskin, 1946). It can occur at any of the sites like sino-atrial junction situated at the exit site of the sinus impulse from SAN to the atrium, atrio-ventricular junction (within AVN, within bundle branch, bundle-Purkinje junction), ectopic atrial junction, ectopic ventricular junction (Schamroth, 1967). Irrespective of the site of its occurrence the fundamental characteristics of Wenckebach phenomenon are typical. It is manifested in ECG as the progressive prolongation of AV conduction time and is terminated by a P wave blocked from entering the ventricles. Clinical reasons for Wenckebach block are digitalis poisoning, infective myocarditis, myocardial infarction, or lesions in the conduction system (Decherd and Ruskin, 1946) when it is occurs at the normal sinus rate. Wenckebach phenomenon is also considered as a protective homeostatic mechanism as it converts the rapid supraventricular rates into safe and physiologically effective ventricular contraction rates (Dubin, 2003). It is also produced when right atrium is stimulated at rates much greater than SA nodal rates (Lister et al., 1965; Damato et al., 1972).

Several hypotheses have been proffered to explain Wenckebach periodicity in AVN. Hoffman's hypothesis (Hoffman et al., 1959) of decremented conduction states that decreased ionic current causes action potential to be delayed progressively along the path of conduction. Rosenblueth (1958) states that the discrete conduction delay which develops at the junction between cells in the N region and NH cells forms the basis of Wenckebach periodicity.

Wenckebach periodicity can happen because of reduced conductivity, changes in the refractory period of the adjacent cells, high atrial pacing rates, change in the excitability of the nodal cells due to channel dysfunctions, medication, or increased vagal activity (Zipes et al., 1983). Because of any of these factors if AP of the preceding cell encroaches into the refractory period of the succeeding AV nodal cells it can cause Wenckebach periodicity. Most of the causes for Wenckebach periodicity are reversible. If the condition is asymptomatic and does not cause a hemodynamic compromise, no treatment is required; but if symptomatic, treatment, including pacing, is required (Epstein et al., 2008).

**Figure 8:**
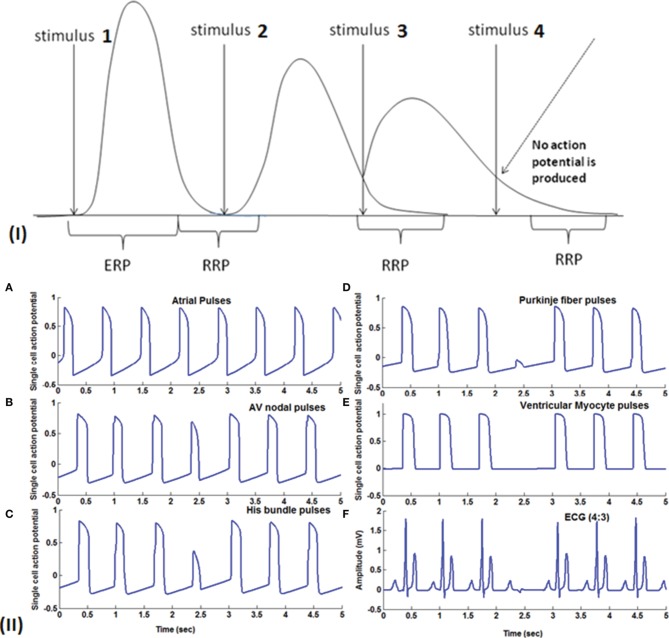
**(I)** Theory of Wenckebach mechanism (4:3): Stimulus 1 causes a normal action potential (AP), stimulus 2 which falls within the RRP (Relative refractory period) causes reduction in the amplitude, reduction in the slope of the upstroke and increase in the duration of AP. Stimulus 3 also falls in the early RRP which produces a further weaker signal with longer APD and this causes stimulus 4 to fall within the ERP (early refractory period) where the cells do not respond to the stimulus. **(II) (A)** Atrial pulses; **(B–D)** Shows gradual reduction in the amplitude of the third signal due to decrease in gap junction conductance and finally in **(E)** fourth pulse failed to stimulate the ventricular myocardial cells causing Wenckebach block 4:3. **(F)** 4:3 Wenckebach block.

In Figure 8, Wenckebach periodicity is explained on the lines proposed by Hoffman's hypothesis (Hoffman et al., 1959) of decremented conduction. If the second stimulus occurs within the relative refractory period (RRP) of the preceding action potential, because of any of the aforementioned reasons, Wenckebach periodicity occurs. When AV nodal cells are stimulated in RRP, the cells would not have regained the normal resting condition. Action potential produced at this state has the following characteristics: it has slope and peak lesser and the APD is also increased (Zipes et al., 1983; Alex et al., 2006), and the conduction velocity is lower. This causes the third stimulus to strike earlier on the down slope of the AP still in RRP and produces a weaker potential with more extended APD than the second one. This causes the fourth stimulus to arrive during the early refractory period (ERP) which does not produce any AP causing a block at this point. This explains the blockage of a beat with periodicity of 4:3, i.e., out of four pulses only three are passed through. Clinical studies reveal that there exist a number of factors that produce AV conduction sequences in Wenckebach block. They are duration of total refractoriness (ERP + RRP), duration of RRP, contour of the recovery curve, conduction time after complete recovery, atrial rate (P-P interval) (Decherd and Ruskin, 1946). Sino-atrial Wenckebach second degree block is also reported and studied to assess its prognostic value. It has been considered as a precursor for the development of high degree sinus exit block (Dąbrowski et al., 2007).

The same phenomenon can occur at high atrial rates and supraventricular tachyarrhythmia also.

In both cases atrial rate is high and this causes a phase shift to be produced among adjacent cells at any anatomic junction causing the impulse to fall in the RRP and then into the ERP causing a pulse to be blocked from reaching the ventricles. But if the phase variations induced are higher because of higher atrial rate and poor coupling across the junctions, it causes type II block causing the pulse to be passed at the ratio X:1. The above conditions are considered as a homeostatic protection mechanism of the AVN which occurs at higher atrial firing rates. But if Wenckebach phenomenon occurs at the normal pacing rates this is mainly because of the increase in APD due to the pathology of the AV nodal cells.

**Figure 9:**
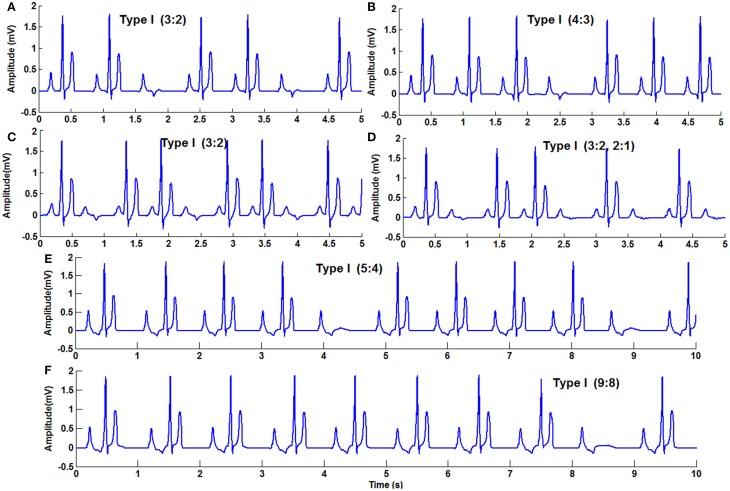
**Second Degree block Type 1 (Wenckebach block) (A) 3:2 block with normal heart rate, (B) 4:3, (C) 3:2 with high atrial rate, (D) varying AV block 3:2,2:1, (E) 5:4, (F) 9:8**.

In WHM2D, Wenckebach periodicity is obtained by varying the excitability and refractory period of the AV nodal cells. The same phenomenon can occur by varying these parameters for bundle fibers and Purkinje fibers. By varying these parameters specific to the cell with moderate GJC (if strong coupling exists the effect of these variations are not seen), it introduces phase shift in the adjacent cells as the rate of recovery of these cells are different. This causes a reduction in the conduction time and the refractory period is prolonged. When one of the atrial pulses falls in the ERP of the cell it results in a block. The ratio (3:2, 4:3, or 5:4) depends on the phase shift induced by the variation in the excitability and difference in the intrinsic frequency of oscillations of the cells at the junction. In the WHM2D the Wenckebach periodicity is observed in different instances of simulation. It is observed when the excitable parameter *v*_*eq*_ is varied for the adjacent cells in the cardiac conduction system, among the cells in the AVN (at the junction between the oscillatory and excitable cells in the AVN), at the junction between AVN and bundle of His and at the junction between the bundle of His and Purkinje cells. It is also observed when the APD of these cells is varied in the junction between two different types of cells, by varying the GJC across the cells while simulating RSA and WPW syndrome. These small changes in APD, GJC, and frequency of oscillations induce phase variations among adjacent oscillators and Wenckebach periodicity is observed (Figure 9). Simulation of Wenckebach block (4:3) and corresponding propagation in the 2D whole heart model is given in Supplementary Material Video 4.

*Mobitz Type II block*. Even though Mobitz in 1924 classified second degree AV block into type I and type II based on the PR interval before and after the blocked P wave, still the distinction between the genesis and prognosis of these two type blocks is not very clear (Langendorf and Pick, 1968; Barold and Hayes, 2001). Type I block, where progressive PR prolongation ends up in a blocked P wave, is assumed to be a functional abnormality and considered reversible (Silverman et al., 2004). Type II block is characterized by constant PR interval with sudden dropped beats in the ratio of X:1, where X is the number of atrial pulses and corresponding to X pulses only one ventricular complex is transmitted. This is assumed to be caused because of structural abnormality and generally believed to occur below AVN (Silverman et al., 2004). But some studies show that it can occur above AVN also (Spear and Moore, 1971), and in such cases QRS complex is narrow (Issa et al., 2012). Compared to Type I, Type II requires attention, and mostly recommended for pacemaker as the structural changes that produced block are irreversible (Epstein et al., 2008).

**Figure 10:**
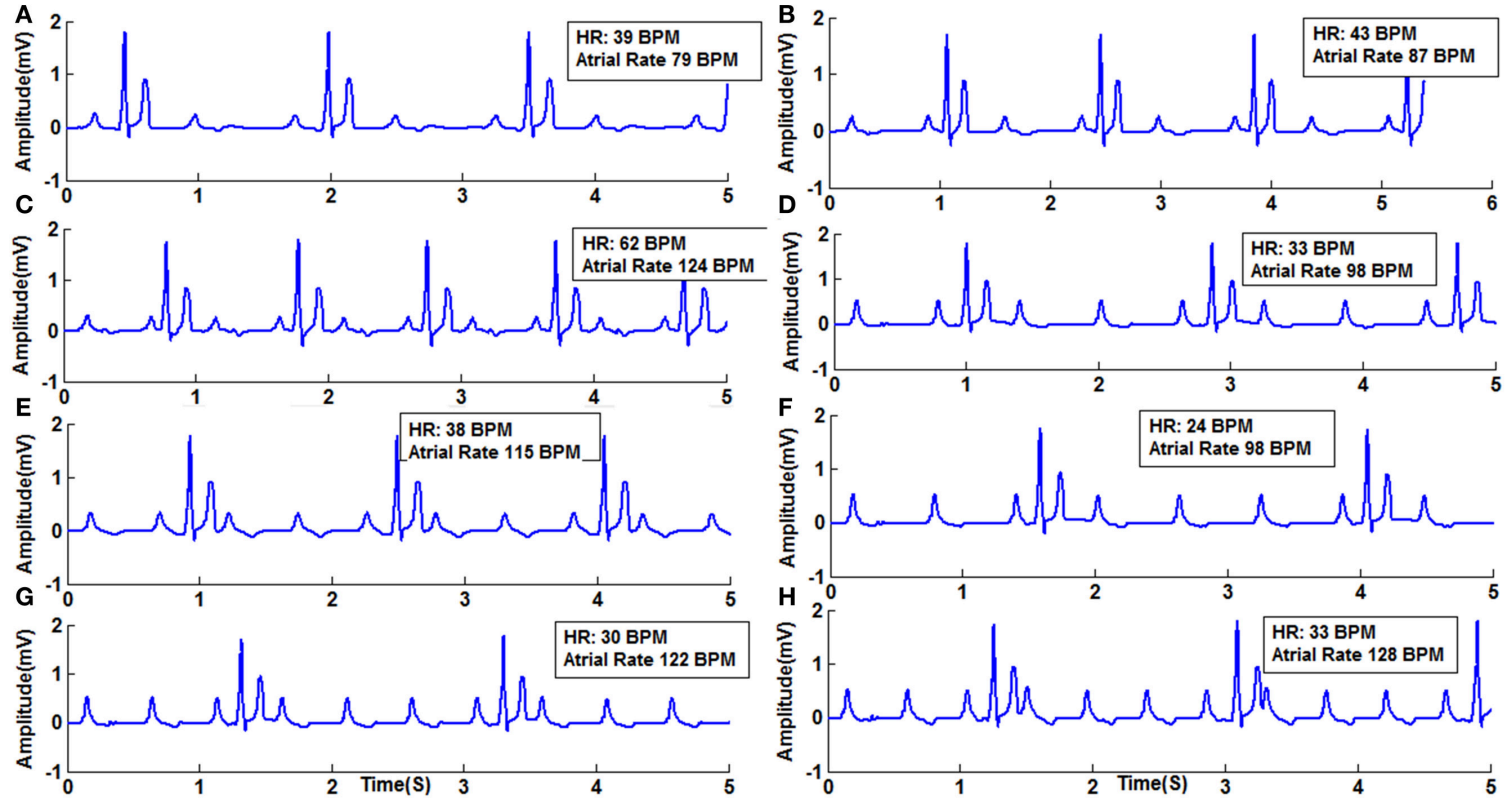
**Second Degree Type II Block (Mobitz) (A) Type II (2:1) with atrial rate 79 BPM, (B) Type 2 (2:1) with atrial rate 87 BPM, (C) Type 2 (2:1) with atrial rate 124 BPM, (D) Type 2 (3:1) with atrial rate 98 BPM, (E) Type 2 (3:1) with atrial rate 115 BPM, (F) Type 2 (4:1) with atrial rate 98 BPM, (G) Type 2 (4:1) with atrial rate 122 BPM, (H) Type 2 (4:1) with atrial rate 128 BPM**.

**Figure 11:**
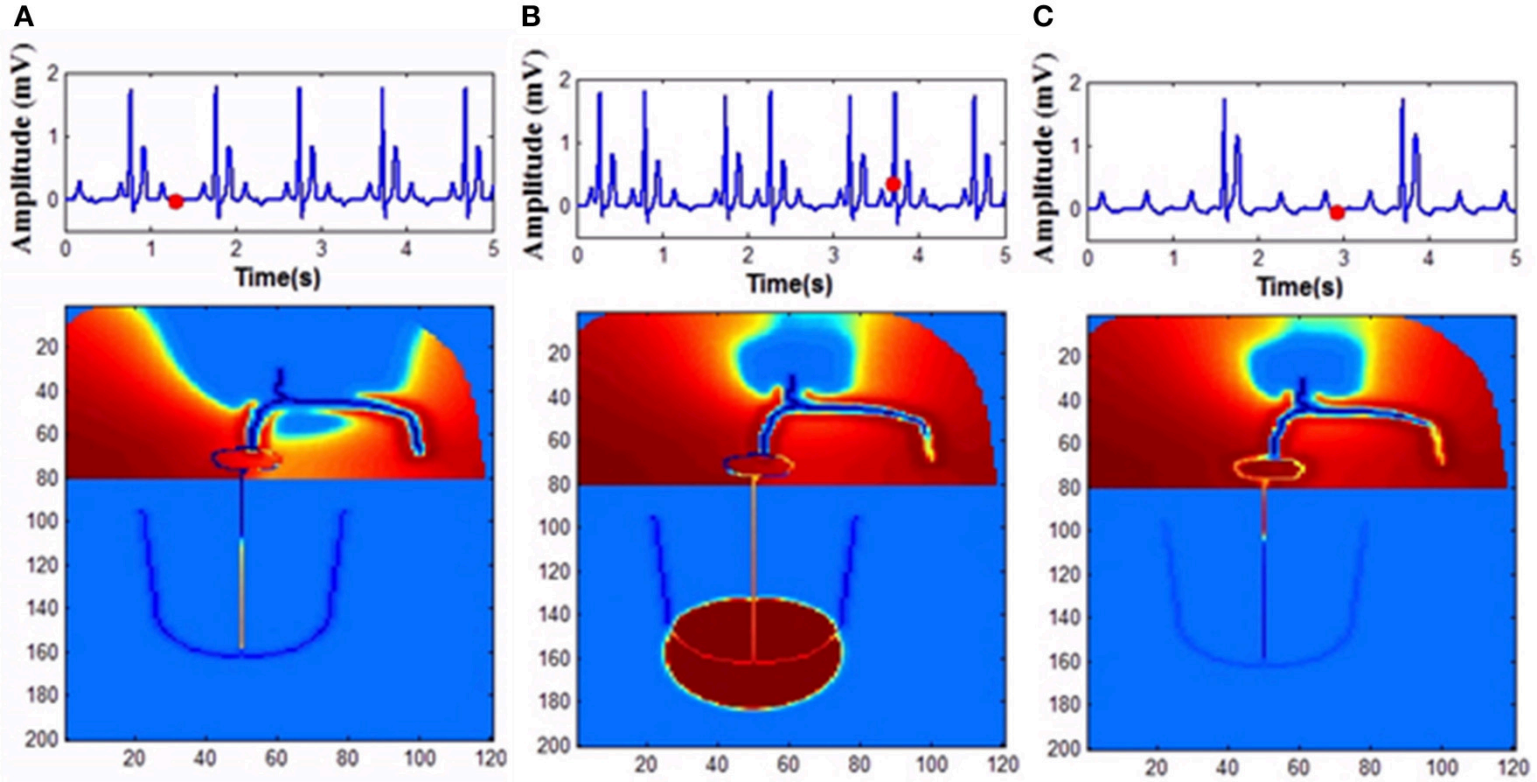
**Second degree block (A) 2:1 block, (B) Wenckebach type I (3:2) block, (C) Mobitz Type II block**.

Mobitz Type II block is simulated in the model by varying the GJC across junction between two different types of cells. Type II blocks of different percentages are obtained by decreasing GJC at the junction between AV nodal cells and bundle cells or bundle cells and Purkinje cells or in the bundle cells. In this case uncoupling among the cells is done by decreasing the conductance value. Simulation of second degree type II (Mobitz) block and corresponding propagation in the 2D whole heart model is given in Supplementary Material Video 5. Various percentages of second degree type II block is shown in Figure 10. Figure 11 shows the spread of impulse corresponding to 2:1 block, Type I, and Type II second degree blocks.

##### Three oscillator model

**Figure 12:**
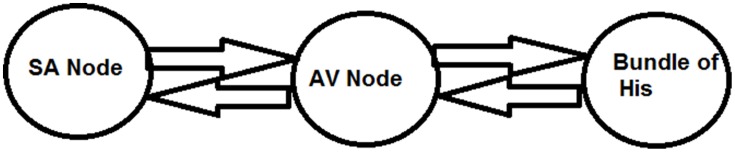
**Three oscillator model**.

To have an easy understanding of the difference between Type I and Type II second degree blocks, we also consider a simple a three oscillator model. Three Fitzhugh-Nagumo (FN) oscillators, representing SAN, AVN, and His bundle cells are connected bidirectionally with GJC as shown in Figure 12. In Table 3 the values used for coupling the oscillators, excitability parameter associated with each oscillator and frequencies of oscillation are given. The oscillators are set to frequencies of higher atrial rate and normal atrial rate. In both the cases, second degree blocks are observed for varying values of excitability and coupling strength. In a three oscillator model, it is easy to pinpoint the parameters which actually cause the block. The first section shows high atrial rate and Wenckebach periodicity is observed by varying the excitability parameter of the bundle of His cells. As the excitability is reduced the percentage of block is also increased. Type II block is obtained by varying the coupling strength. As the coupling strength is decreased higher percentage of block is obtained. This concept complies with the physiological data, as Wenckebach periodicity is not a structural abnormality and can be caused by some temporary phenomenon that varies the excitability of the cells. But Type II can be related to structural changes like uncoupling incase of infarctions and they need more attention than type I block (Silverman et al., 2004). The same concept is implemented in the 2DWH model to reproduce Type I and Type II blocks.

**Table 3:** **Three oscillator model showing parameters selected for Type I and Type II (Second Degree) blocks**.

**BPM (b)**			**Excitability (*****V***_*****eq*****_**)**			**Coupling strength (γ)**				**Phenomena observed**
**SAN**	**AVN**	**Bundle of His**	**SAN *v*_*eq*1_**	**AVN *v*_*eq*2_**	**Bundle of His *v*_*eq*3_**	**SAN γ_12_**	**AVN**		**Bundle of His γ_32_**	
							**γ_21_**	**γ_23_**		
182	70	40	0.23	0.12	**0.23**	0.003	0.04.	0.005	0.05	Wenckebach (4:3)
			0.23	0.12	**0.2**	0.003	0.04	0.005	0.05	Wenckebach (3:2)
			0.23	0.12	**0.16**	0.003	0.04	0.005	0.05	Wenckebach (2:1)
			0.23	0.12	0.12	0.003	0.04	0.02	**0.05**	Mobitz (3:1)
			0.23	0.12	0.12	0.003	0.04	0.02	**0.03**	Mobitz (4:1)
			0.23	0.12	0.12	0.003	0.04	0.02	**0.02**	Mobitz (6:1)
			0.23	0.12	0.12	0.003	0.04	0.02	**0.01**	Mobitz (8:1)
76	56	34	0.23	0.13	**0.2**	0.004	0.04	0.005	0.05	Wenckebach (4:3)
			0.23	0.13	**0.18**	0.004	0.04	0.005	0.05	Wenckebach (3:2)
			0.23	0.13	**0.14**	0.004	0.04	0.005	0.04	Wenckebach (2:1)
			0.23	0.13	0.12	0.004	0.04	0.005	**0.007**	Mobitz (5:1)
			0.23	0.13	0.12	0.004	0.04	0.005	**0.01**	Mobitz (4:1)
			0.23	0.13	0.12	0.004	0.04	0.005	**0.02**	Mobitz (3:1)

The bundle of His is a collection of heart muscle cells specialized for electrical conduction that transmits the electrical impulses from the AV node.

Because of the variations in intrinsic factors like reduction in GJC or the variation in the refractory period of the adjacent cells causes the impulse to fall in the ERP and can result in a block. In addition to this when the atrial rate is too high also impulses are conducted in the ratio X:1, where X is the number of atrial pulses and corresponding to X atrial contractions, ventricular contraction takes place only once.

(12) $$\begin{array}{l} f\left(v1\right)=v1\left(v1-a1\right)\left(1-v1\right)\\ dv1∕dt=f\left(v1+v_{eq1}\right)-f\left(v_{eq1}\right)-w1+\gamma_{12}\left(v2-v1\right)\\ dw1∕dt=b1\left(v1-cw1\right)\\ f\left(v2\right)=v2\left(v2-a2\right)\left(1-v2\right)\\ dv2∕dt=f\left(v2+v_{eq2}\right)-f\left(v_{eq2}\right)-w2+\gamma_{21}\left(v1-v2\right)\\ +\;\gamma_{23}\left(v3-v2\right)\\ dw2∕dt=b2\left(v2-cw2\right)\\ f\left(v3\right)=v3\left(v3-a3\right)\left(1-v3\right)\\ dv3∕dt=f\left(v3+v_{eq3}\right)-f\left(v_{eq3}\right)-w3+\gamma_{32}\left(v2-v3\right)\\ dw3∕dt=b3\left(v3-cw3\right) \end{array}$$

where *v*_*eq*1_ denotes the excitability of SA nodal cells

*v*_*eq*2_ denotes the excitability of AV nodal cells

*v*_*eq*3_ denotes the excitability of bundle of His cells

γ_12_ Coupling strength from SAN to AVN

γ_21_ Coupling strength from AVN to SAN

γ_23_ Coupling strength from AVNtobundle of His cells

γ_32_ Coupling strength from bundle of His cells to AVN

The values of the excitability and coupling strength for various conditions are given in Table 3.

##### 2:1 AV block

Second degree block is classified into two types, type I (X:X-1) and type II (X:1). 2:1 (50%) block without impaired hemodynamics may belong to type I which can be a functional abnormality and does not require treatment. But 2:1 type II block can be a precursor of complete block as it results from structural impairment and may require immediate attention. Exercise testing is done to differentiate these two types as exercise increases the sympathetic drive and reduces the vagal tone (Issa et al., 2012). As a result, if the block is Wenckebach type (type I), conduction increases to 3:2 (67%) or 4:3 (75%) or even higher. But the block existing is type II, conduction decreases to 3:1 (33%) or 4:1 (25%) or even to lower rates.

**Figure 13:**
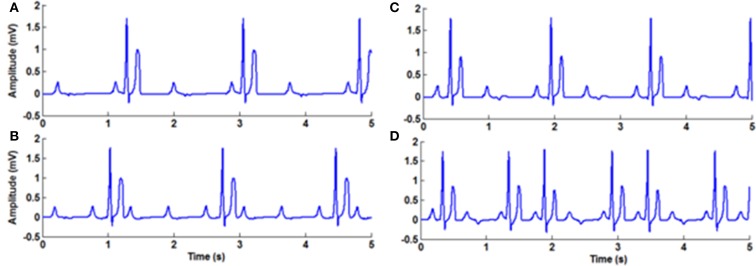
**2:1 Block (A) 2:1 Mobitz Type II Block (normal atrial rate 69 BPM), (B) 3:1 Mobitz Type II Block obtained by increasing the atrial rate (105 BPM) with the same GJC parameters, (C) 2:1 Mobitz Type I (Wenckebach) Block atrial rate 78 BPM) (B) 3:2 Mobitz Type II (Wenckebach) Block obtained by increasing the atrial rate (117 BPM) with the same parameters**.

In the WHM2D the same phenomenon is observed. When ECG is simulated by reduced GJC (0.1 μS) among the bundle cells, 2:1 block is observed with normal atrial rate (69 BPM) and when the atrial rate is increased to 105 BPM, 3:1 block is observed. and it was confirmed that the block present belongs to Mobitz type II. In another case of 2:1 block as observed with high conductance among His bindle cells (0.9 μS) and with atrial rate 78 BPM. With the same parameters atrial rate is increased to 117 BPM and 3:2 block is obtained confirming that the block present in the model is type I Wenckebach block (Figure 13).

##### Third degree block

Complete AV block occurs when atrial pulses are blocked from entering the ventricles. This can happen at different anatomic areas: at the AV node, bundle, or bundle branches. In complete (third degree) block AV dissociation occurs and therefore both atria and ventricles contract asynchronously. Atrial contractions continue to occur at the normal rate. However, since pacemaker cells exist in the ventricular system also, the ventricles start pacing at their own rhythm (30-40 BPM). PR interval appears varying in the ECG signal recorded as the synchronism is lost between atria and ventricles, but PP interval and RR interval will remain fixed. If the complete block exists for a few days, with reduction in cardiac output, it is recommended for Class I pacemaker implantation (Epstein et al., 2008). The causes of third degree block can be myocardial infarction, calcification of aortic valve, infections, neuromuscular diseases, collagen vascular diseases, congenital defects in conduction system and drugs like digoxin, amiodarone, verapamil (Khan, 2005). It also occurs when surgical correction is done for congenital heart problems (Gross et al., 2006).

**Figure 14:**
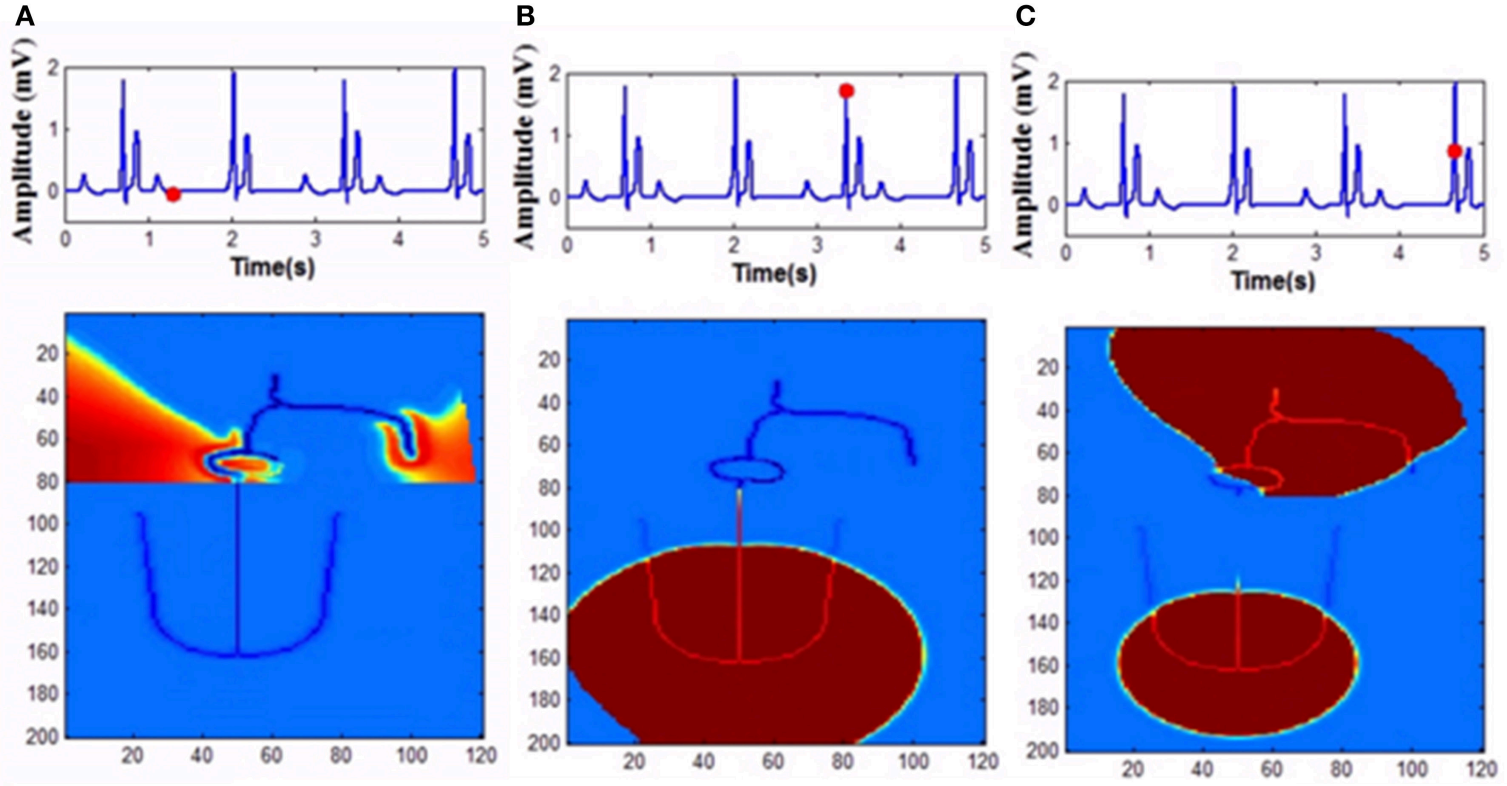
**Complete heart block (A) Atrial activity fails to stimulate ventricular activity as the GJC at the junction between the AVN and bundle cells is low (1 pS), (B) As the co-ordination is lost ventricles are firing its own, (C) Both atrial and ventricular contractions are happening simultaneously too**.

In the model, for simulating complete heart block condition, GJC is made very low at the junction between AVN and bundle of His or among the bundle cells. Atrial impulses are produced at the normal rate. If the block is at the AV junction, escape impulses are produced from the His bundle cells (60-40 BPM) or if the block is at the bundle cells, ventricles contract corresponding to impulses from the Purkinje system (40-20 BPM) (Figure 14).

#### Wolf-Parkinson-White (WPW) syndrome

In normal functioning of the heart, impulses from atria are passed through AVN, the only electrical connection to the ventricles. But in pre-excitation syndromes, bypass tracts (BT) or accessory pathways emerge between atria and ventricles, in addition to AVN. Because of BTs, ventricles will be excited before impulses from atria reach ventricles through AVN, as there is no conduction delay in the accessory pathway. BTs are formed as remnants of continuity between atrial and ventricular myocardium during the embryological development of AV annuli (Sethi et al., 2007; Issa et al., 2012). Even though this anomaly is present at birth, the onset of arrhythmic conditions varies and except for the appearance of delta wave and a short PR interval in the ECG, some people do not present any symptoms. In that case it is known as WPW pattern, and if this bypass tract causes supraventricular tachyarrhythmia because of reentry into the atria, then it is termed as WPW syndrome (Deal et al., 1985). BTs are thin strands of working myocardial cells which allow bidirectional (anterograde and retrograde) and unidirectional (anterograde or retrograde) conduction (Issa et al., 2012). Other arrhythmias like atrial tachycardia, atrial fibrillation and AVN re-entry tachycardia (AVNRT) can coexist with WPW syndrome. Electrophysiological mapping is done to distinguish WPW syndrome with AVNRT. When the refractory period of the AVN, bundle cells, as well as that of the cells in the accessory path way, is reduced, it results in atrial fibrillation by continuous triggering of the atria by anterograde conduction through the accessory pathway and retrograde conduction through the bundle fibers and AVN. When the cells are repetitively triggered causing multiple conduction into the ventricles in case of atrial tachyarrhythmia, it can also trigger ventricular fibrillation (Douglas and Zipes, 2009). When the accessory pathway does not limit the number of impulses triggering the ventricles from the atria due to atrial fibrillation, patients with WPW syndrome may develop ventricular fibrillation (VF) which can result in sudden cardiac death (SCD) (Prystowsky et al., 1987). For patients with WPW syndrome, electrophysiological testing is done to assess the refractory period of the myocardial cells in the accessory pathway. If they are shorter to avoid the risk of development of VF and SCD, catheter ablation of AP is done with radiofrequency current (Kuck and Schlüter, 1991; Kuck et al., 1991).

**Figure 15:**
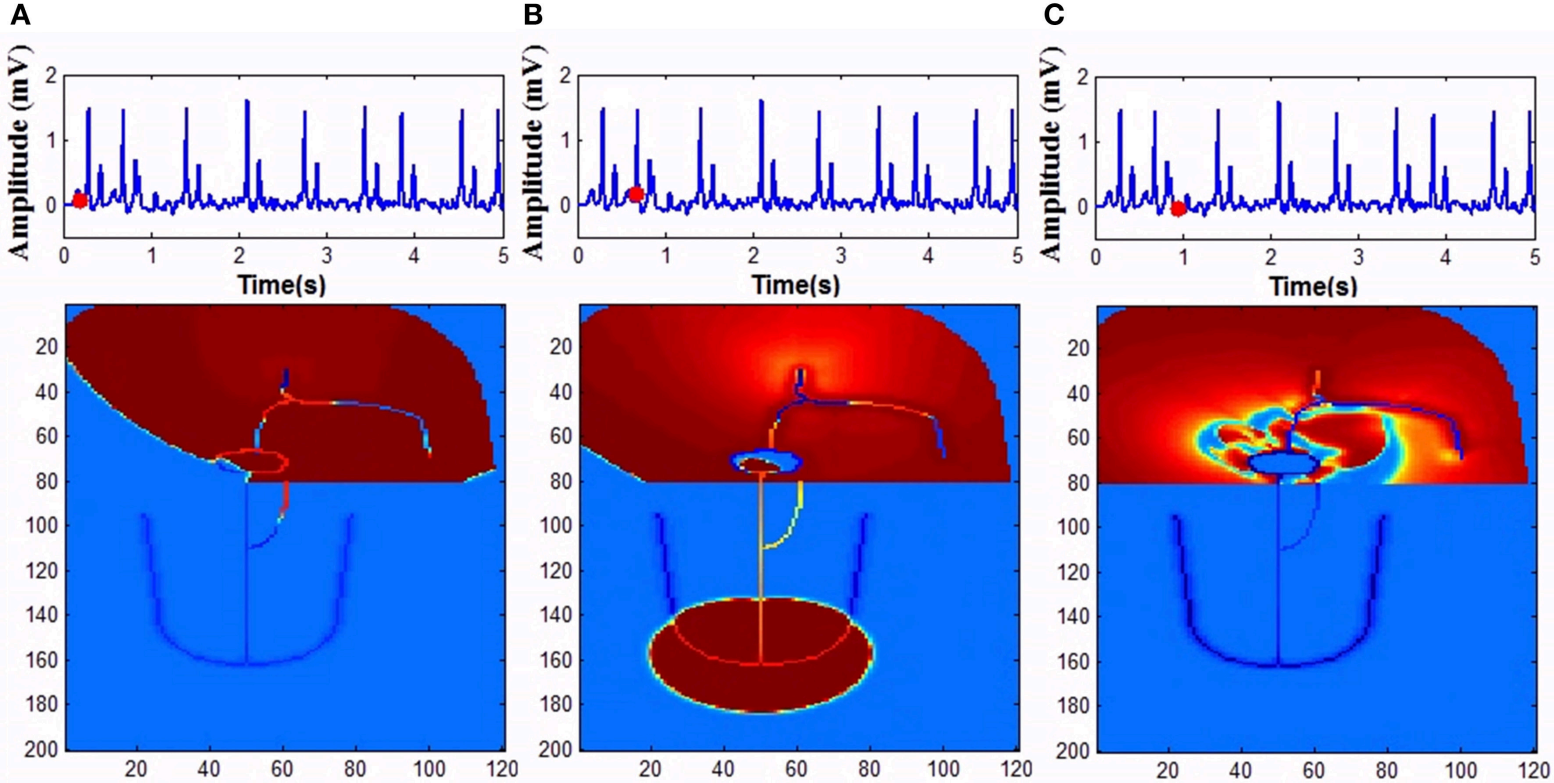
**Wolf Parkinson White syndrome (A) Anterograde conduction through the bypass tract triggers ventricles earlier (B) Retrograde conduction though the bundle fibers and AV node causes retriggering of the atria**. **(C)** Retriggering in atria causes tachyarrhythmia.

In WHM2D, an accessory pathway is introduced connecting the atria to the bundle of His through ventricular musculature, bypassing the AVN. The cells in the accessory pathway are implemented by the FN two-variable model in the excitable mode. High GJC is assigned to the cells in the path so that the accessory pathway has faster conduction compared to than that of AVN. Because of high GJC, impulses through the accessory path reach ventricles faster than through the AVN. When the refractory period of the cells in the accessory pathway is longer, ECG appears normal except for the small change in the PR interval and the appearance of a delta wave in the onset of the QRS complex. When the refractory period is made shorter for the cells in the accessory pathway and AVN, impulses that propagate via retrograde conduction through bundle fibers can reach AVN causing retriggering. This further triggers the cells in the accessory pathway as they can also be retriggered by the fast pulses from atria and the process goes on (Figure 15). This can cause the onset of atrial tachyarrhythmia. If this condition prolongs for longer durations, it can cause VF (Prystowsky et al., 1987). Simulation of WPW pattern and WPW syndrome and corresponding propagation in the 2D whole heart model is given in Supplementary Material Video 6 and Video 7.

## Conclusion

Computational modeling at the whole heart level with ECG simulation can give powerful insights into understanding of the electrophysiological function of heart in normal and arrhythmic conditions. Computational modeling of the electrical activity of heart presents several key challenges: the complex 3D geometry of heart, spatial representation at the resolution of the heart, heterogeneity of the cells (autorhythmic cells and excitable cells), heterogeneity in action potential shape and duration, non-linearity of the ODE describing the ionic currents and the number of variables involved, stiffness of the membrane dynamics (time scale different for different phases of the action potential—fast upstroke (phase 1) and slowly varying plateau (phase 2) and repolarization process (phase 3), rotational anisotropy of fiber orientation in different regions, inhomogeneity of gap junction conductance in different regions etc. The complexity of the model and the computational load can become impractically high if all these factors are taken into consideration while modeling. The choice of the modeling approach, dimension and structure of the model should be determined by the purpose of the model, level of quantitative details required and should involve an intelligent balance between the computational load and modeling objectives. The simplified approach used in the model is sufficient to capture the cell dynamics and able to explain many of the fatal and non-fatal arrhythmias.

Very few computational models correlate with ECG and arrhythmias. The one dimensional model for AV conduction using rabbit cell model explains fast and slow conduction in AV node and the filtering properties of AVN in case of atrial fibrillation (Inada et al., 2009). But the arrhythmic conditions are not explained in the context of whole heart activity. The 2DWH model explains all conduction abnormalities and the filtering properties of AV node using reduced models with APD variations and GJ coupling. The bidomain model of ventricles developed by Boulakia et al simulates 12 lead ECG with APD heterogeneity and anisotropy of muscle fibers (Boulakia et al., 2010). Since atria are not included in the model, P wave is not present and the model is stimulated by external activation signal. Even though there is evidence of heterogeneity throughout the ventricular myocardium (Noble and Cohen, 1978; Antzelevitch, 2001), APD heterogeneity (transmural) is assumed only for left ventricles. The only arrhythmia that was explained by the model is bundle blocks in both left and right branches. 2DWH model explains cardiac activity at the whole heart level and all the intrinsic properties of the cardiac electrical activity is taken into account. The autorhythmic cells in the specialized conductive system takes care of the rhythmic activity of the cardiac cycle, GJ coupling, and APD heterogeneity modulates the spread of AP in the myocardium and variation in the APD and poor GJ coupling results in arrhythmia conditions.

Source model based interactive software ECGSIM, which simulates ECG signal for different arrhythmic conditions, does not consider the GJC for the propagation of signal in the myocardium. Various intervals and segments of ECG in normal and abnormal conditions cannot be correlated with GJC or APD heterogeneity (van Oosterom and Oostendorp, 2004; van Dam et al., 2010; van Oosterom et al., 2011).

There are several issues associated with the reaction diffusion systems which are used to evaluate the electrical activity using bidomain or monodomain approaches. They are the complexity of the domain (geometric complexity of heart), spatial representation at the resolution of heart which determines the spatial discretization process (usually cellular automata is used) and stiffness of the membrane dynamics (which limits the step size to be very small). Also the conductivity tensor used in the diffusion equation depends on the spatial position, rotational anisotropy and inhomogeneity of electrical conductivities present in the medium (Ying, 2005). This creates problems in the computation of the bidomain model as the solutions may not converge if the anisotropy is strong and the conductivity tensor is rapidly changing. Conductivity tensor is assumed to be homogeneous in the model and the individual distribution of GJC cannot be taken into account. Enormous computational load is required to generate realistic ECG waveforms in normal and arrhythmia conditions using bidomain approaches as homogenization is assumed to the discrete nature of cardiac cells and most of the arrhythmias are generated at cell level (changes in frequency of autorhythmic cells, APD variations) or intercellular interactions (variation on anisotropy, GJC).

The proposed WHM2D can be used to analyze the generation of arrhythmias, both fatal and non-fatal in relation to the ECG signal generated. Arrhythmias are classified based on the changes in the ECG signal. It is a standard approach for assessing the cardiac function. Based on the experimental studies on several animal species the spread of impulse in the normal propagation of ECG and causes of each arrhythmia are understood. In the 2D model the relationship of each type of arrhythmia to the parameters (GJC, APD, rate of oscillation of SA Node) is demonstrated in detail. In the arrhythmias tachycardia, bradycardia, and respiratory sinus arrhythmia the rate of the oscillators in the conduction system are varied according to the control factor from ANS. In sinus pause, the temporary impairment of SA nodal cells causes the electrical activity to come to stand still for a few seconds. When the causes are withdrawn recovery occurs with a junctional escape and thereafter normal activity continues. Atrio-ventricular conduction blocks are also simulated with a clear discussion of the underlying mechanism. Simulated activation sequence of WPW syndrome shows the conduction through accessory pathways and how atrial fibrillation is triggered when APD of these cells become shorter.

Use of computational models gives more insight into the mechanisms by which arrhythmias are generated. Ideally, a model should as simple as possible to explain the complex dynamics of the heart. This simple 2D model can explain many of the complex arrhythmia microscopically as we can see the cellular dynamics and macroscopically as changes in ECG and 2D propagation (Balakrishnan et al., 2014). In comparison to the available computational models with ECG generation the proposed WHM2D can simulate many more fatal and non-fatal arrhythmias with less computational complexity. It takes only 30 min of computation time for simulating 1 s of cardiac activity in a desktop machine with i5 Intel processor @ 2.8 GHz. Most of the cardiac models are based on ionic models and realistic geometries at the resolution of normal heart. They take too much time, and extensive hardware and software resources for simulating few seconds of cardiac activity. Cardiac arrhythmia research project (CARP) takes 6.4 h for simulating 200 ms of cardiac activity in a 64 processor machine (Mitchell, 2010). Many architectures based on GPU are developed to reduce the computation time required for simulating bidomain equations (Bordas et al., 2009; Yu et al., 2010).

Limitations of the model: Ideally a whole-heart model must be a 3D model since the real heart is three-dimensional. Therefore, 2D whole heart model carries certain inherent limitations. One feature of activation propagation dynamics possible in a 3D model—a propagating wave that can circulate around the heart—is inherently disallowed in a 2D model. But the objective of the present study is to explore how much of cardiac dynamics can be captured with a simple 2D model. The sizes of the atria and ventricular chambers in the model are not exactly to scale of the real heart. In the future, some of the anatomical features of the WHM2D (location of SA node and AV node, the exact geometry of the Purkinje network etc) can be formally optimized to achieve a closer fit to the ECG waveform. Uniform connectivity is assumed for every cell in the model (each cell is connected to its adjacent eight neighbors through GJC which varies according to the type of the cell). However, the number of neighbors of a cell depends on the cell type in the real heart. Since the model uses reduced two-variable cell models the ionic current variations that underlie arrhythmogenesis cannot be taken into account. But simplified whole heart models of the kind proposed here are useful in providing valuable insights into cardiac dynamics. They reveal the components that are crucial for achieving various aspects of ECG waveform, distinguishing them from those that are not indispensable.

Future work involves the study of pacing effects in the model which can explain how the arrhythmia conditions can be corrected by stimulating pulses. Another interest is to explain the phenomenon of cardiac memory which is caused by the external electrical activation and persists long after the presentation of stimulus is terminated (Rosenbaum et al., 1982). Work has been done to support the hypothesis that the adaptive dynamic cardiac gap junction is responsible for the intriguing phenomenon of cardiac memory using a 2D network (Chakravarthy and Ghosh, 1997; Krishnan et al., 2008; Sachdeva et al., 2010). We wish to demonstrate cardiac memory in the WHM2D by incorporating adaptive gap junctions and external pacing.

## Author contributions

Mrs. MB (PhD student). Dr. VC (Guide). MD, PhD. SG (Coguide). MB, VC designed and conceived the model, MB implemented the model, analyzed the results, all the authors interpreted the result and prepared the manuscript.

## Funding

The research was supported by Indian Institute of Technology Madras.

### Conflict of interest statement

The authors declare that the research was conducted in the absence of any commercial or financial relationships that could be construed as a potential conflict of interest.

## Supplementary material

Video 1 **Simulations of normal ECG from the 2DWH model**. The generation of ECG along with the propagation of cardiac impulse in the 2D model is shown.

Video 2 **The generation of respiratory sinus arrhythmia along with the propagation of cardiac impulse in the 2D model is shown**. Here the SA node impulse generation is modulated with respiratory rhythm.

Video 3 **Sinus Pause occurs when the SA nodal pulses are not generated for a short duration of time (greater than 2 s) The generation of sinus pause along with the propagation of cardiac impulse in the 2D model is shown**.

Video 4 **Simulation of second degree type I (Wenckebach block) is shown along with the propagation of cardiac impulse**. 4:3 block is simulated by varying gap junction conductance in AV node.

Video 5 **Simulation of second degree type II (Mobitz block) is shown along with the propagation of cardiac impulse**. 3:1 block is simulated by decreasing gap junction conductance in His bundle.

Video 6 **Simulation of Wolf-Parkinson-White (WPW) pattern is shown along with the propagation of cardiac impulse**. An accessory pathway other than AV node is created which bypasses AV node. Since the action potential duration of cells in the bypass tract and AV node are longer retriggering is not happening. Except for the small change in QRS ECG is normal.

Video 7 **Simulation of Wolf-Parkinson-White (WPW) syndrome is shown along with the propagation of cardiac impulse**. An accessory pathway other than AV node is created which bypasses AV node. The action potential duration of cells in the bypass tract and AV node are made shorter and retriggering occurs in atria producing AVNRT.
